# The HDAC10 instructs macrophage M2 program via deacetylation of STAT3 and promotes allergic airway inflammation

**DOI:** 10.7150/thno.82535

**Published:** 2023-06-19

**Authors:** Yu Zhong, Tong Huang, Jiewen Huang, Jingyun Quan, Guomei Su, Zhilin Xiong, Yingying Lv, Shihai Li, Xianwen Lai, Yuanyuan Xiang, Qu Wang, Lianxiang Luo, Xiao Gao, Yiming Shao, Jing Tang, Tianwen Lai

**Affiliations:** 1 Institute of Respiratory Diseases, Affiliated Hospital of Guangdong Medical University, Zhanjiang 524001, China.; 2 Department of Respiratory and Critical Care Medicine, The First Dongguan Affiliated Hospital, Guangdong Medical University, Dongguan 523121, China.; 3 The Marine Biomedical Research Institute, Guangdong Medical University; The Marine Biomedical Research Institute of Guangdong Zhanjiang, China.; 4 The Intensive Care Unit, The First Dongguan Affiliated Hospital, Guangdong Medical University, Dongguan 523121, China.; 5 Department of Anesthesiology, Affiliated Hospital of Guangdong Medical University, Zhanjiang 524001, China.

**Keywords:** HDAC10, STAT3, asthma, airway inflammation, macrophage.

## Abstract

**Background:** Perturbation of macrophage homeostasis is one of the key mechanisms of airway inflammation in asthma. However, the exact mechanisms remain poorly understood.

**Objectives:** We sought to examine the role of histone deacetylase (HDAC) 10 as an epigenetic regulator that governs macrophage M2 program and promotes airway inflammation in asthma, and to elucidate the underlying mechanisms.

**Methods:** Peripheral blood and airway biopsies were obtained from healthy individuals and asthmatic patients. Asthma was induced by exposure to allergen in mice with myeloid-specific deletion of* Hdac10* (*Hdac10^fl/fl^*-*LysMCre*) mice. HDAC10 inhibitor Salvianolic acid B (SAB), STAT3 selective agonist Colivelin, and the specific PI3K/Akt activator 1,3-Dicaffeoylquinic acid (DA) were also used in asthmatic mice. For cell studies, THP1 cells, primary mouse bone marrow derived macrophage (BMDMs) were used and related signaling pathways was investigated.

**Results:** HDAC10 expression was highly expressed by macrophages and promoted M2 macrophage activation and airway inflammation in asthmatic patients and mice. *Hdac10^fl/fl^*-*LysMCre* mice were protected from airway inflammation in experimental asthma model. *Hdac10* deficiency significantly attenuated STAT3 expression and decreased M2 macrophage polarization following allergen exposure. Mechanistically, HDAC10 directly binds STAT3 for deacetylation in macrophages, by which it promotes STAT3 expression and activates the macrophage M2 program. Importantly, we identified SAB as a HDAC10 inhibitor that had protective effects against airway inflammation in mice.

**Conclusions:** Our results revealed that HDAC10-STAT3 interaction governs macrophage polarization to promote airway inflammation in asthma, implicating HDAC10 as a therapeutic target.

## Introduction

Asthma is a common respiratory disease, which characterized by chronic airway inflammation, hyper responsiveness, and airway remodeling. The incidence of asthma increases by 50% every 10 years on average worldwide 1. However, the mechanisms underlying the pathogenesis of asthma are not completely understood and urgently require in-depth investigation.

Lung macrophages are crucial sentinels in host lung defense and play crucial role in the maintenance of immune regulation, pathogen clearance, and homeostasis 2,3. The pathogenesis of asthma involves perturbed lung homeostasis response to environmental factors exposure such as house dust mites (HDM), cigarette smoke, and lipopolysaccharide (LPS). Macrophages are classified into two categories: classically activated phenotype (M1) and alternatively activated phenotype (M2) 4. M1-polarized macrophages are activated under the action of IFN-γ. M2 macrophages are classically activate by IL-4 or IL-13. We, and others, have shown previously that macrophages, particularly M2 macrophages, play an important role in the pathogenesis in asthma, such as bronchial hyperactivity, airway inflammation, and remodeling 5-7. Although perturbation of macrophage homeostasis is associated with the pathogenesis of asthma, the underlying mechanisms remain elusive. Genes encoding transcription factors of the signal transducer and activator of transcription (STATs) family are involved in the cell activation 8. STAT3 is associated to the occurrence and development of immune diseases, infectious diseases and tumors. Recent studies have shown that STAT3 is a critical determinant of M2 macrophage polarization and the expression of macrophage M2 markers (Arg1, Fizz1, and Ym1) 9,10. However, the regulatory mechanisms that repress STAT3 expression, and thereby inhibit macrophage M2 program, remain largely unknown.

Emerging evidence suggests that epigenetic modifications are crucial for the pathogenesis of asthma. Acetylation is a widely occurring epigenetic modification of proteins that are involved in diverse biological processes 11. The acetylation status is maintained by an intricate balance of histone deacetylase (HDAC) and histone acetyltransferase (HAT) 12. HDAC10 is a class IIb HDAC that plays critical roles in regulating cellular processes, genomic stability, cancer progression, cells autophagy, and stress response via its epigenetic functions 12-16. However, whether and how HDAC10 regulates macrophage homeostasis in asthma has not been deciphered.

Here, we found that HDAC10 expression was highly expressed by macrophages and played a critical role in maintaining macrophage homeostasis by controlling M2 macrophage activation through deacetylating STAT3. Mechanistically, HDAC10 directly interacts with STAT3 and targets STAT3 for acetylation, by which it promotes STAT3 expression to increase the macrophage M2 program. Both genetic and pharmacological inhibition of HDAC10 protect against airway inflammation in asthmatic mice, suggesting that the HDAC10/STAT3 axis is a potential target for treatment of asthma.

## Results

### HDAC10 expression was elevated in asthmatic patients and mice

**Figure 1:**
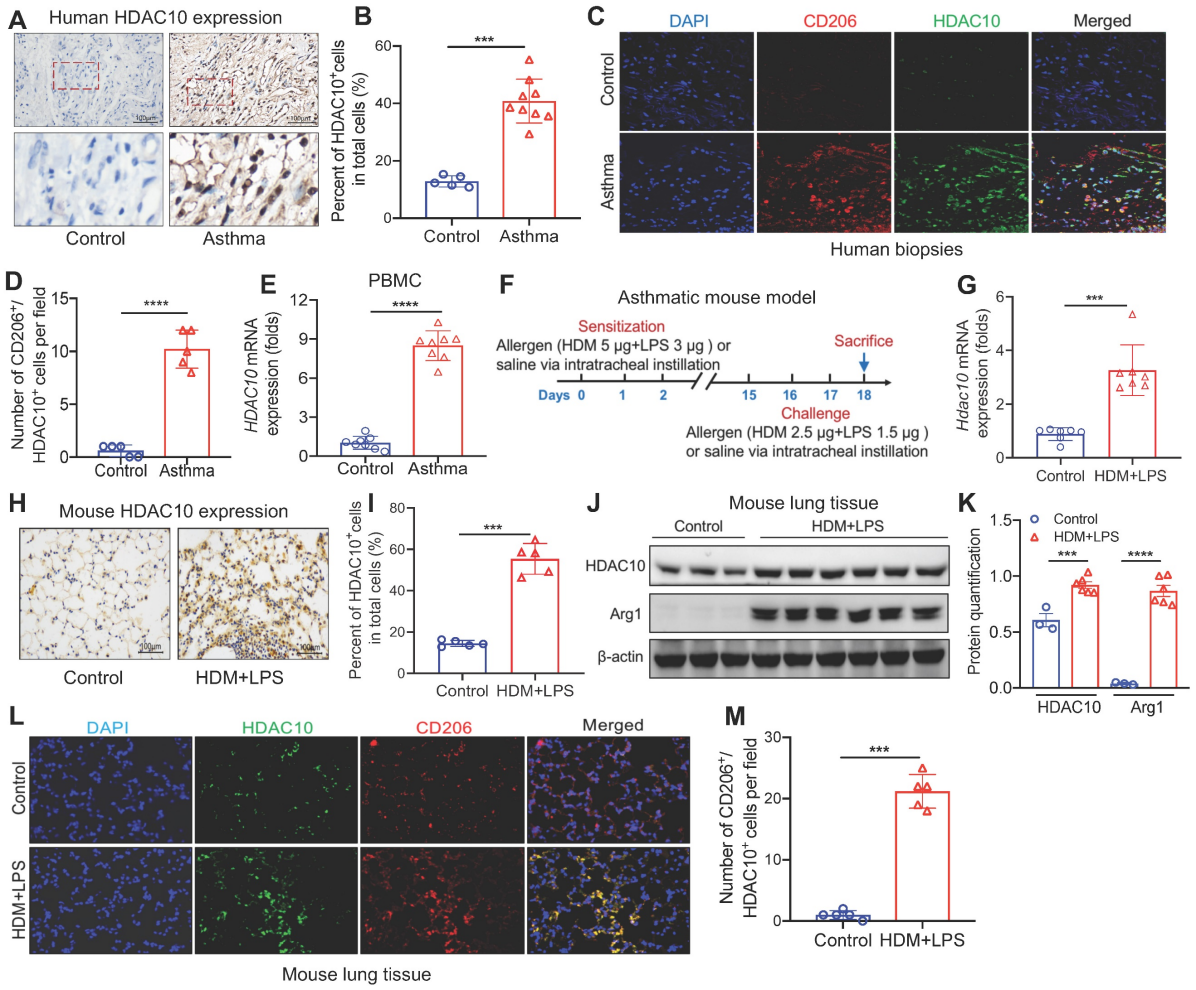
** Analysis of HDAC10 expression in asthmatic patients and mice. (A, B)** Representative IHC staining of HDAC10 in the airway from asthmatic patients. Images were captured at ×400 magnification and quantification of IHC was done by using Image J software. **(C, D)** Representative results for coimmunostaining of HDAC10 and CD206 in the lung sections from patients with asthma. Images were captured at ×400 magnification and quantification of IHC was done by using Image J software. **(E)** Peripheral blood mononuclear cells (PBMC) were isolated from peripheral blood samples of study subjects. The* HDAC10* expression in PBMC was analyzed by using quantitative RT-PCR **(**qRT-PCR**)**. **(F)** Schematic illustrating an established house dust mite **(**HDM**)**/lipopolysaccharide** (**LPS**)**-induced asthma mouse model (n = 6-8 for each group). **(G)** qRT-PCR analysis of the expression of *Hdac10* in mouse lung tissue. Results were normalized to those of the gene encoding β-actin, and relative expression was calculated by the change-in-threshold method. **(H, I)** Representative IHC images of HDAC10 in lung tissue of *Hdac10^fl/fl^* mice following allergen induction. Images were captured at ×400 magnification and quantification of IHC was done by using Image J software. **(J, K)** Western blot analysis of HDAC10 and macrophage M2 marker Arg1 expression in lung tissues of *Hdac10^fl/fl^* mice following allergen induction and quantification was done by using Image J software. **(L, M)** Results for coimmunostaining of HDAC10 and macrophage M2 marker CD206 in allergen-induced lung sections. Images were captured at ×400 magnification and quantification was done by using Image J software. Data are shown as means ± SEM. ^***^P < 0.001 and ^****^P < 0.0001 versus Control (unless otherwise noted) by two-tailed unpaired Student's t test. Data are representative of three independent experiments with similar results **(A, C, H, and L)** or are from three independent experiments **(G and J)**. See also Figure S1.

We first detected HDAC10 expression in the airway biopsies from asthmatic patients (Table S1). HDAC10 was highly expressed in the airway biopsies from asthmatic patients compared with normal controls (Figure 1A-B). Using coimmunostaining of M2 macrophage maker CD206, we found that HDAC10 was mainly localized in M2 macrophages (Figure 1C-D). Using quantitative RT-PCR (qRT-PCR), we next detected the expression of HDAC10 from human normal and asthmatic peripheral blood mononuclear cells (PBMCs) (Table S2). Compared with normal controls, the levels of *HDAC10* were also significantly increased in asthmatic patients (Figure 1E).

To further determine the role of HDAC10 in the pathogenesis of asthma, we detected HDAC10 expression in the lung tissue in an experimental model of asthma (Figure 1F). As assessed by qRT-PCR, we observed that *Hdac10* was significant increased compared with controls (Figure 1G). Those results were confirmed by immunohistochemistry (IHC) and Western blotting (Figure 1H-K). Consistent with human data, HDAC10 abundance was also mainly localized in macrophages in the lung tissue of asthmatic mice (Figure 1L-M). Moreover, we found that HDAC10 expression was induced in the THP1 cells and bone marrow-derived macrophages (BMDMs) in allergic stimuli (Figure S1A-F). Together, these findings suggested that HDAC10 was highly expressed in M2 macrophages and potentially promoted airway inflammation, which also prompted us to further explore the precise role of macrophage HDAC10 in airway inflammation process in asthma.

### *Hdac10* deficiency protected mice against allergen-induced airway inflammation

**Figure 2:**
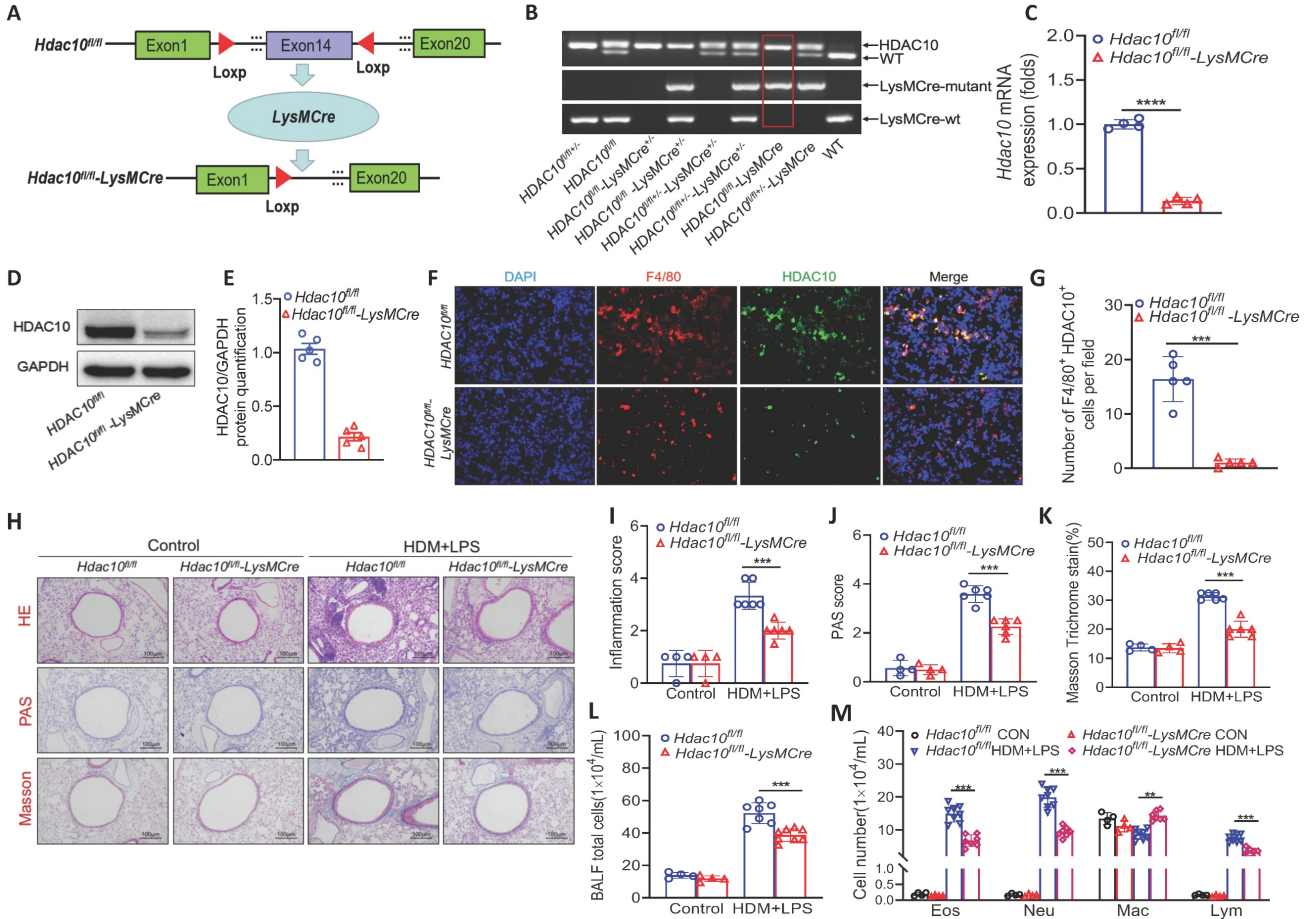
***Hdac10* deficiency protected mice against allergic airway inflammation. (A)** Schematic illustrating the genetic approach used to generate macrophages-conditional knockout of *Hdac10* (*Hdac10^fl/fl^*-*LysMCre*) mice. **(B)** *Hdac10* deficiency was confirmed by assessing genomic DNA. **(C-E)** *Hdac10* deficiency was assessed in BMDMs from *Hdac10^fl/fl^* and *Hdac10^fl/fl^*-*LysMCre* mice using qRT-PCR and Western blotting analysis. Quantification was done by using Image J software. **(F, G)** Representative result for coimmunostaining of F4/80 and HDAC10 in the lung sections from *Hdac10^fl/fl^* and *Hdac10^fl/fl^*-*LysMCre* mice. Images were captured at ×400 magnification and quantification was done by using Image J software. **(H-M)** Total BAL fluid (BALF) cells, differential cell counts, and histologic analysis of the lung sections were performed with hematoxylin and eosin staining to visualize inflammatory cell recruitment from *Hdac10^fl/fl^* and *Hdac10^fl/fl^*-*LysMCre* mice treated with allergen. Images were captured at ×200 magnification and quantification was done by using Image J software. Data are shown as means ± SEM (n = 4-6 mice/group). ^**^P < 0.01 and ^***^P < 0.001 versus Control (unless otherwise noted) by two-tailed unpaired Student's t test or one-way ANOVA, followed by Tukey's multiple comparisons test. Data are representative of three independent experiments with similar results **(F and H)** or are from three independent experiments **(C, D, L and M)**. See also Figure S2.

To dissect how HDAC10 exerts its function during allergic airway inflammation, we generated mice with macrophage-specific *Hdac10* knocked out (*Hdac10^fl/fl^*-*LysMCre* mice) and their littermates (*Hdac10^fl/fl^* mice) (Figure 2A). All mice were genotyped with PCR (Figure 2B). The absence of HDAC10 from bone marrow macrophages was verified by Western blotting and qRT-PCR (Figure 2C-E). The absence of HDAC10 colocalization with macrophages was observed in the lung sections of* Hdac10^fl/fl^*-*LysMCr*e mice using immunofluorescence (IF) staining (Figure 2F-G). The *Hdac10^fl/fl^*-*LysMCre* and *Hdac10^fl/fl^* mice were exposed to allergen according to an asthma model as described in Figure 1F. Hematoxylin & eosin (HE) staining, periodic acid Schiff (PAS) staining, and peribronchial trichrome (Masson) staining were reduced in HDM/LPS-exposed *Hdac10^fl/fl^*-*LysMCre* mice (Figure 2H-K). The total inflammatory cells, neutrophils, eosinophils, and lymphocytes in bronchoalveolar lavage fluid (BALF) were reduced in allergen-exposed *Hdac10^fl/fl^*-*LysMCre* mice (Figure 2L-M). The mRNA and protein expression of CXCL1 and CXCL2 were lower in the lung tissues and BALF of *Hdac10^fl/fl^*-*LysMCre* mice than in that of *Hdac10^fl/fl^* mice exposed to allergen (Figure S2A-F). Consistent with these findings, HDAC10 deficiency suppressed the production of CXCL1 and CXCL2 in allergen-induced BMDMs (Figure S2G-H). Together, our data support that *Hdac10* deficiency in macrophages protects mice from allergen-induced airway inflammation.

### *Hdac10* deficiency attenuated M2 macrophage activation

**Figure 3:**
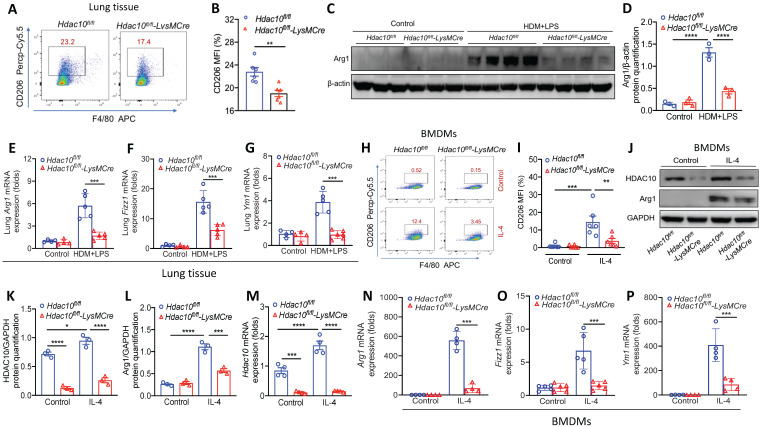
***Hdac10* deficiency inhibited the macrophage M2 program. (A**, **B)** Flow cytometry analysis (BD Biosciences) of macrophages derived from lung tissues of *Hdac10^fl/fl^* and *Hdac10^fl/fl^*-*LysMCre* mice after allergen induction. Quantification of CD206^+^ cells was analyzed with FlowJo (TreeStar). **(C, D)** Western blotting analysis of macrophage M2 marker Arg1 expression in the lung homogenates of *Hdac10^fl/fl^* and *Hdac10^fl/fl^*-*LysMCre* mice after allergen induction. Quantification was done by using Image J software. **(E-G)** qRT-PCR analysis of macrophage M2 markers* Arg1*, *Fizz1* and *Ym1* mRNA expression in the lung tissue of *Hdac10^fl/fl^* and *Hdac10^fl/fl^*-*LysMCre* mice after allergen exposure. **(H**, **I)** Flow cytometry analysis (BD Biosciences) of macrophage M2 marker CD206 expression in BMDMs following IL-4 stimulation. Quantification of CD206^+^ cells was analyzed with FlowJo (TreeStar). **(J-L)** Western blotting analysis of HDAC10 and Arg1 expression in the BMDMs after IL-4 induction. Quantification was done by using Image J software. **(M-P)** qRT-PCR analysis was conducted for *Hdac10* mRNA and macrophage M2 markers* Arg1*, *Fizz1*, and *Ym1* mRNA expression in the BMDMs after IL-4 induction. Data are shown as means ± SEM (n = 4-6 mice/group). ^**^P < 0.01 and ^***^P < 0.001 versus Control (unless otherwise noted) by two-tailed unpaired Student's t test or one-way ANOVA, followed by Tukey's multiple comparisons test. Data are representative of three independent experiments with similar results **(C and J)** or are from three independent experiments **(A, E-H, and M-P)**. See also Figure S3.

The above results revealed prompted us to further explore the precise role of HDAC10 on M2 macrophage polarization in the airway inflammation process during asthma. The flow cytometry showed that the proportion of M2 macrophages (F4/80^+^CD206^+^) in the lung of *Hdac10^fl/fl^*-*LysMCre* mice were lower than that of *Hdac10^fl/fl^* mice following allergen exposure (Figure 3A-B). Consistently, Western blotting showed that M2 marker Arg1 was reduced in *Hdac10^fl/fl^*-*LysMCre* mice as compared with the *Hdac10^fl/fl^* mice (Figure 3C-D). Moreover, M2 markers* Arg1*, *Fizz1* and *Ym1* demonstrated similar results (Figure 3E-G).

To further dissect the mechanism of *Hdac10* regulates the M2 program of macrophages, we generated BMDMs from *Hdac10^fl/fl^* and *Hdac10^fl/fl^*-*LysMCre* mice and then stimulated with interleukin-4 (IL-4). The average fluorescence intensity of CD206 in *Hdac10^fl/fl^* BMDMs induced by IL-4 was significantly higher than that of *Hdac10^fl/fl^*-*LysMCre* BMDMs (Figure 3H-I). The HDAC10 protein expression was significantly increased in *Hdac10^fl/fl^* BMDMs upon IL-4 induction, while Arg 1 was decreased in *Hdac10^fl/fl^*-*LysMCre* BMDMs compared with *Hdac10^fl/fl^* BMDMs following IL-4 stimulation (Figure 3J-L). The mRNA expression of *Hdac10* and M2 markers *Arg1*, *Fizz1*, and *Ym1* revealed similar results (Figure 3M-P).

The above data supported the hypothesis that *Hdac10* deficiency protected mice against allergen-induced airway inflammation via attenuating the M2 program. To address this assumption, adoptive transfer experiments were performed as described in Methods. Briefly, BMDMs prepared from* Hdac10^fl/fl^* and *Hdac10^fl/fl^*-*LysMCre* donor mice and then stimulated with IL-4. The above BMDMs were adoptively transferred into macrophage-depleted wildtype (WT) recipient mice after allergen induction. We found that macrophages were almost undetectable in the lung tissue of mice treat with clodronate liposome (Figure S3A-B). Macrophage-depleted WT mice received *Hdac10^fl/fl^* BMDMs treated with IL-4 exhibited higher M2 markers (*Arg1* and *Ym1*) production like* Hdac10^fl/fl^* mice following allergen challenge. In contrast, macrophage-depleted WT mice received *Hdac10^fl/fl^*-*LysMCre* BMDMs treated with IL-4 appeared similar to *Hdac10^fl/fl^*-*LysMCre* mice after allergen administration, with lower M2 markers (Arg1 and Ym1) production (Figure S3C-D).

Consistent with these results, HE staining suggested that the adoptive transfer of *Hdac10^fl/fl^* BMDMs restored allergen-induced airway inflammation in macrophage-depleted WT mice (Figure S3E-F). As shown in Figure S3G-J, the cytokine secretion remained decreased in allergen-challenged macrophage-depleted WT mice compared with controls after transfer of* Hdac10^fl/fl^-LysMCre* BMDMs. Collectively, this data defined a unique function of *Hdac10* in macrophages in the regulation of asthmatic airway inflammation.

### Depletion of *Hdac10* repressed PI3K/Akt signaling

**Figure 4:**
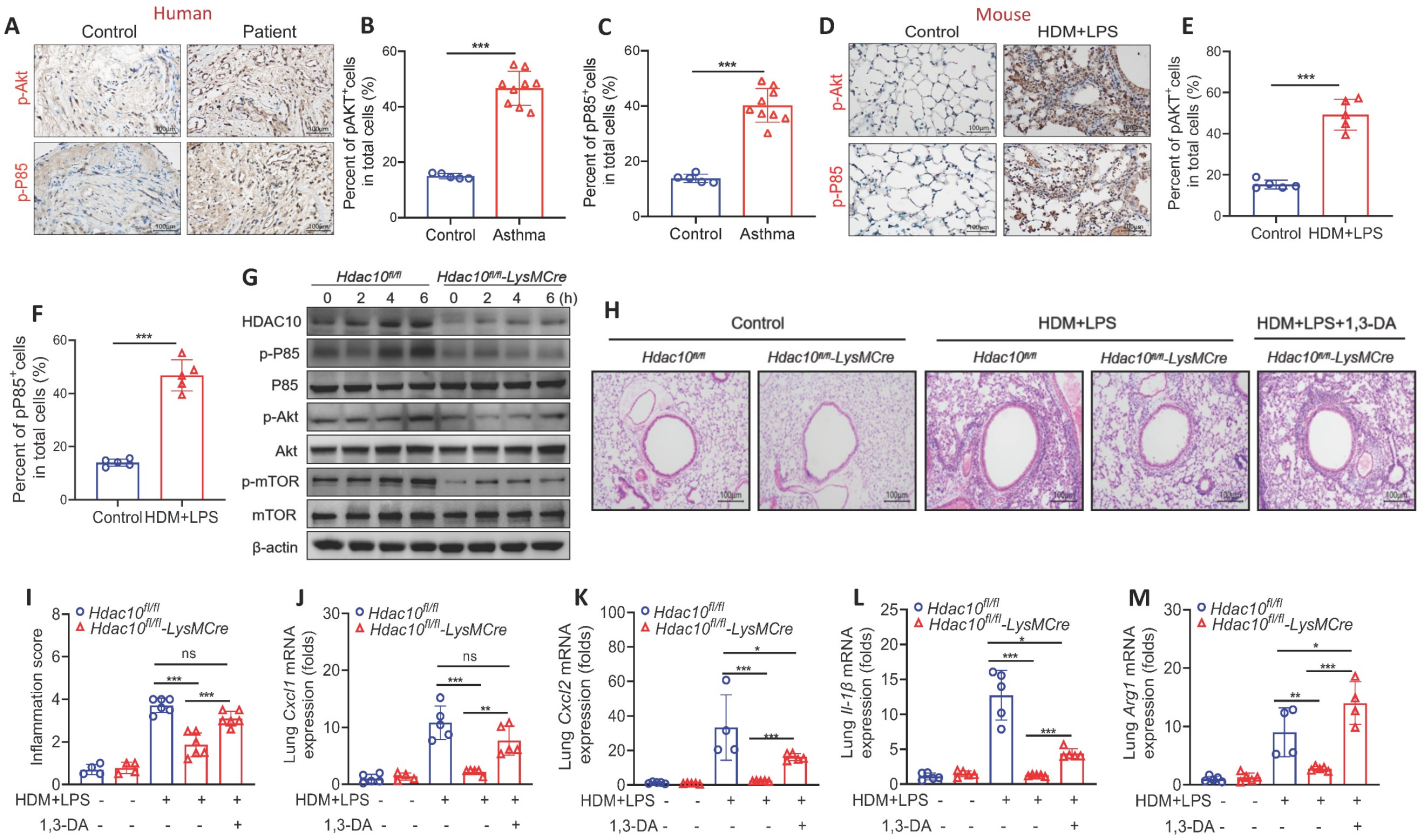
***Hdac10* deficiency attenuated allergen-induced PI3K/Akt signaling in macrophages. (A-C)** Representative IHC staining of p-Akt and p-P85 in the airway from asthmatic patients. Images were captured at ×400 magnification and quantification of IHC was done by using Image J software. **(D-F)** Representative IHC staining of p-Akt and p-P85 in the lung tissue from asthmatic mice. Images were captured at ×400 magnification and quantification of IHC was done by using Image J software. **(G)** Western blotting analysis of p-Akt, p-mTOR and p-P85 expression in the BMDMs from *Hdac10^fl/fl^* and *Hdac10^fl/fl^*-*LysMCre* mice after allergen exposure. **(H, I)** Representative photomicrographs of lung inflammation expression are shown. Images were captured at ×200 magnification and quantification was done by using Image J software. **(J-L)** The expression of inflammatory cytokines in the lung homogenate of mice was analyzed by using qRT-PCR. **(M)** qRT-PCR analysis was conducted for macrophage M2 markers* Arg1* mRNA expression in the lung homogenate of mice. Data are shown as means ± SEM (n = 4-6 mice/group). ns, not significant, ^*^P < 0.05, ^**^P < 0.01, ^***^P < 0.001, and ^****^P < 0.0001 versus Control (unless otherwise noted) by two-tailed unpaired Student's t test or one-way ANOVA, followed by Tukey's multiple comparisons test. Data are representative of three independent experiments with similar results **(A, D, G, and H)** or are from three independent experiments **(J-M)**. See also Figure S4.

Given the important role of PI3K/Akt signaling played in macrophage M2 polarization induced by IL-4 or IL-13 17,18, we first assessed the expression of PI3K/Akt signaling in asthmatic patients and mice. The expression of phosphorylated P85 (p-P85) and p-Akt was up-regulated in asthmatic patients and mice (Figure 4A-F). We further found that the levels of p-P85, p-Akt and the Akt downstream effector p-mTOR were decreased in *Hdac10^fl/fl^*-*LysMCre* BMDMs compared with* Hdac10^fl/fl^* BMDMs induced by IL-4 stimulation (Figure 4G, Figure S4A-D).

To explore the possible role of PI3K/Akt in mediating the role of HDAC10 in macrophage inflammation, we used the specific PI3K/Akt activator 1,3-Dicaffeoylquinic acid (DA) to activate PI3K/Akt pathway as descried in Figure S4E. Our studies demonstrated that airway inflammation was reduced in allergen-exposed* Hdac10^fl/fl^*-*LysMCre* mice compared with *Hdac10^fl/fl^* mice, but 1,3-DA treatment almost completely reversed these phenomena (Figure 4H-I). Consistent with these findings, 1,3-DA treatment significantly rescued the reduced *Cxcl1*,* Cxcl2*,* IL-1β* and *Arg1* production in the lung tissue of *Hdac10^fl/fl^*-*LysMCre* mice after allergen stimulation (Figure 4J-M). Collectively, our data support that *Hdac10* deficiency repressed PI3K/Akt signaling to attenuate airway inflammation following allergen induction.

### *Hdac10* deficiency suppressed STAT3 to inhibit M2 macrophage program

**Figure 5:**
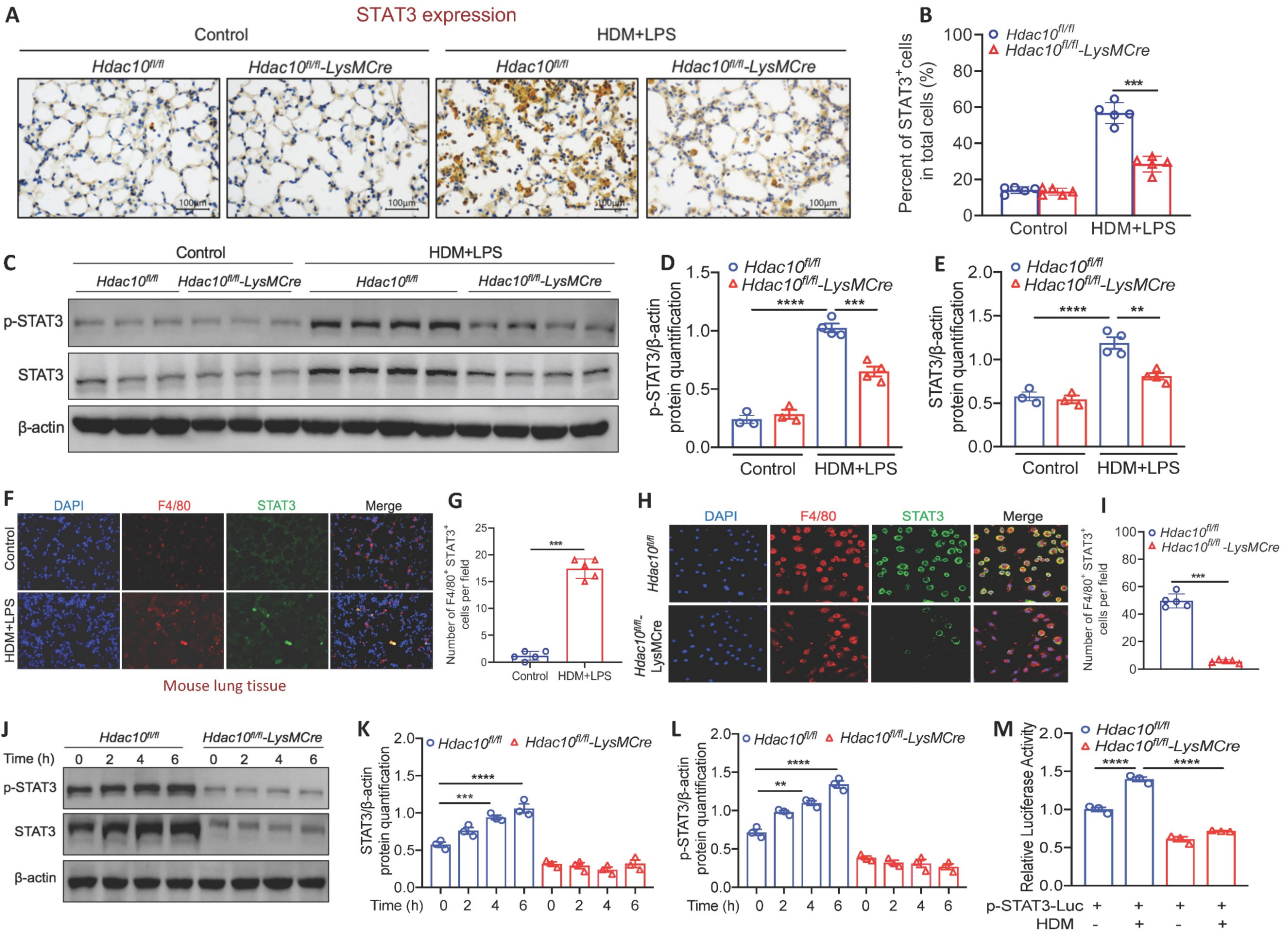
***Hdac10* deficiency suppressed STAT3 to attenuate M2 program. (A, B)** Representative IHC staining of STAT3 in the lung tissue from *Hdac10^fl/fl^* and *Hdac10^fl/fl^*-*LysMCre* mice after allergen induction. Images were captured at ×400 magnification and quantification of IHC was done by using Image J software. **(C, E)** Western blotting analysis of p-STAT3 and STAT3 expression in the lung homogenates of *Hdac10^fl/fl^* and *Hdac10^fl/fl^*-*LysMCre* mice after allergen induction. Quantification was done by using Image J software. **(F, G)** Representative result for coimmunostaining of F4/80 and STAT3 in the lung sections from *Hdac10^fl/fl^* and *Hdac10^fl/fl^*-*LysMCre* mice after allergen induction. Images were captured at ×400 magnification and quantification was done by using Image J software. **(H, I)** Representative result for coimmunostaining of F4/80 and STAT3 in the BMDMs from *Hdac10^fl/fl^* and *Hdac10^fl/fl^*-*LysMCre* mice. Images were captured at ×400 magnification and quantification was done by using Image J software. **(J-L)** Western blotting analysis was conducted for p-STAT3 and STAT3 expression in the BMDMs after allergen stimulation. Quantification was done by using Image J software. **(M)** BMDMs from *Hdac10^fl/fl^* and *Hdac10^fl/fl^*-*LysMCre* mice were transfected with pSTAT3-TA-luc plasmid for 24 h, and then treated with HDM (100 μg/ml) for another 24 h. Luciferase assay was used to detected STAT3-TA-luc fusion protein to asses STAT3 transactivation activity. Data are shown as means ± SEM. ^**^P < 0.01, ^***^P < 0.001, and ^****^P < 0.0001 versus Control (unless otherwise noted) by two-tailed unpaired Student's t test or one-way ANOVA, followed by Tukey's multiple comparisons test. Data are representative of three independent experiments with similar results **(A, C, F, H and J)** or are from three independent experiments **(M)**.

It has been reported that PI3K/AKT pathway serves as an up-stream participant in the STAT3 activation 19. STAT3 plays an important role in the activation of immune cells (e.g., T cells, macrophages, eosinophils) and contributes to the development of asthma 20. Therefore, it promoted us to explore whether *Hdac10* deficiency attenuates macrophages M2 program via STAT3 activation. We first compared STAT3 expression in the lung tissue between *Hdac10^fl/fl^* and *Hdac10^fl/fl^*-*LysMCre* mice following allergen stimulation. IHC staining showed that STAT3 expression was significantly reduced in lung tissue of *Hdac10^fl/fl^-LysMCre* mice compared with *Hdac10^fl/fl^* mice exposed to allergen (Figure 5A-B). Consistent with this result, p-STAT3 and STAT3 in *Hdac10^fl/fl^*-*LysMCre* mice was expressed at a much lower level of protein than in the lung tissue of *Hdac10^fl/fl^* mice after allergen induction (Figure 5C-E). We further found that STAT3 was also mainly localized in macrophages infiltrated in the lung tissue of mice after allergen induction by F4/80 staining (Figure 5F-G). Furthermore, p-STAT3 and STAT3 expression was obviously increased in allergen-induced *Hdac10^fl/fl^* BMDMs, but significantly decreased in allergen-induced* Hdac10^fl/fl^*-*LysMCre* BMDMs (Figure 5H-L). In addition, we expressed a STAT3-TA-luc fusion protein to asses STAT3 transactivation activity by luciferase assay. The activity of STAT3 was significantly augmented in allergen-induced *Hdac10^fl/fl^* BMDMs, whereas it was attenuated in allergen-induced *Hdac10^fl/fl^*-*LysMCre* BMDMs (Figure 5M).

**Figure 6:**
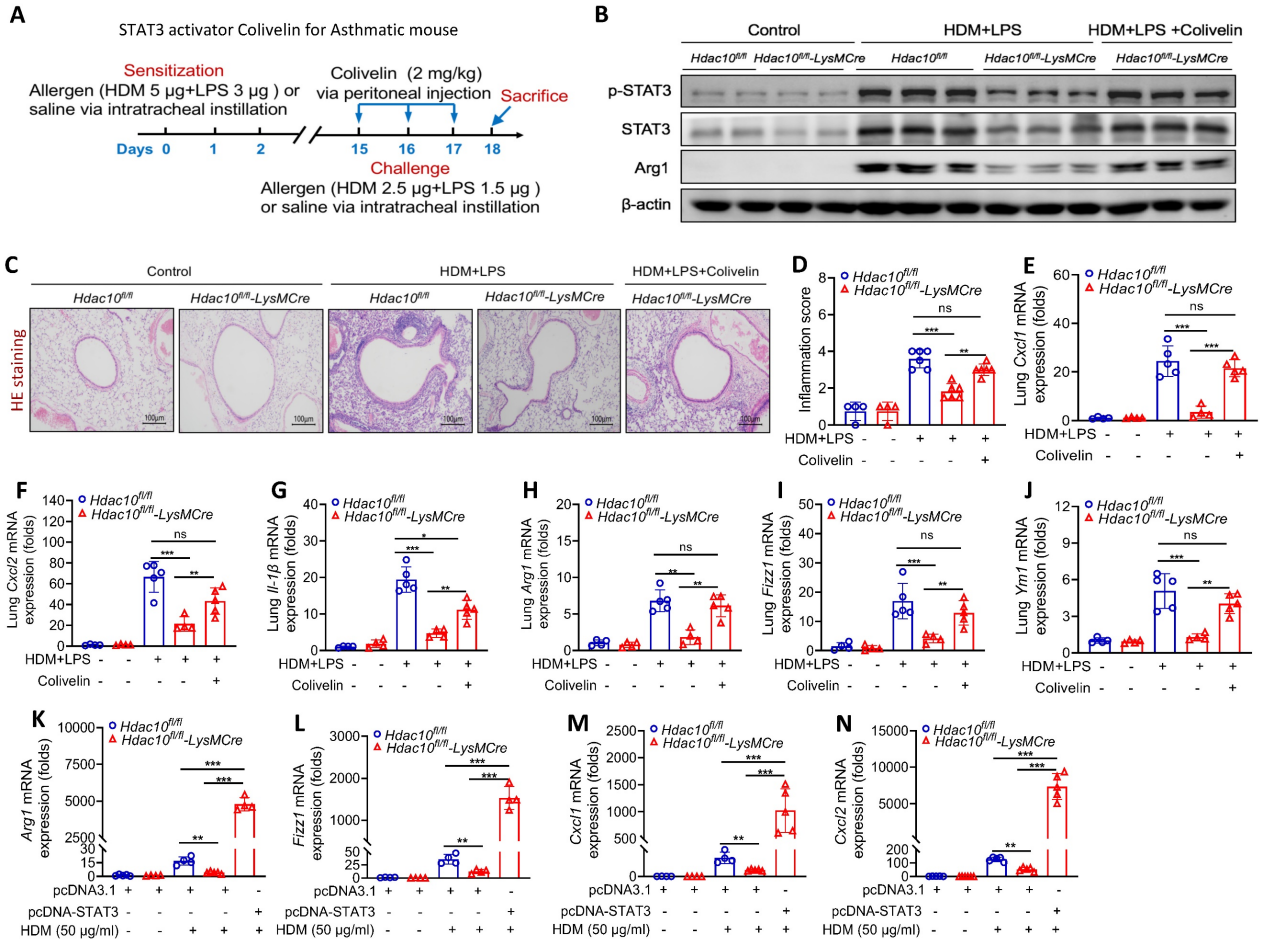
** STAT3 activator Colivelin exacerbated allergic airway inflammation in *Hdac10* deficiency mice. (A)** Schematic overview of experimental design for STAT3 activator Colivelin for asthmatic mouse. **(B)** The expression of p-STAT3, STAT3 and Arg1 in the lung homogenate from mice treated with allergen or Colivelin was analyzed by using Western blotting. **(C, D)** Representative photomicrographs of lung inflammation expression is shown. Images were captured at ×200 magnification and quantification was done by using Image J software. **(E-G)** The expression of inflammatory cytokines in the lung homogenate of mice was analyzed by using qRT-PCR. **(H-J)** qRT-PCR analysis was conducted for macrophage M2 markers* Arg1*, *Fizz1*, and *Ym1* mRNA expression in the lung homogenate of mice. **(K-N)** BMDMs were transfected with Control or STAT3 plasmid for 24 h and then treated with HDM for another 24 h. *Arg1*, *Fizz1*, *Cxcl1*, and *Cxcl2* mRNA expression were assessed by qRT-PCR analysis. Data are shown as means ± SEM (n = 4-6 mice/group). ns, not significant, ^*^P < 0.05, ^**^P < 0.01, ^***^P < 0.001, and ^****^P < 0.0001 versus Control (unless otherwise noted) by one way ANOVA, followed by Tukey's multiple comparisons test. Data are representative of three independent experiments with similar results **(B and C)** or are from three independent experiments **(E-N)**. See also Figure S4.

To further investigate whether STAT3 regulate macrophage M2 polarization in this context, STAT3 selective agonist Colivelin was administered to mice as described in Figure 6A. As predicted, p-STAT3, STAT3 and Arg1 expression were significantly lower in lung tissue of *Hdac10^fl/fl^*-*LysMCre* mice compared with *Hdac10^fl/fl^* mice exposed to allergen, but Colivelin treatment almost completely reversed these phenomena (Figure 6B, Figure S4F-H). Compared with the control treatment, Colivelin treatment increased the inflammatory cells in airway inflammation (HE staining), inflammatory cytokines, and macrophage M2 markers (Arg1, Fizz1, and Ym1) in lung tissue of *Hdac10^fl/fl^*-*LysMCre* mice exposed to allergen (Figure 6C-J). Consistent with these findings, overexpression of STAT3 promoted allergen-induced inflammatory cytokines, and macrophage M2 markers in allergen-induced *Hdac10^fl/fl^*-*LysMCre* BMDMs (Figure 6K-N). Collectively, our data supported that* Hdac10* deficiency suppressed STAT3 to attenuate M2 programming during allergic inflammation.

### HDAC10 directly interacted with STAT3 and targeted STAT3 for deacetylation

**Figure 7:**
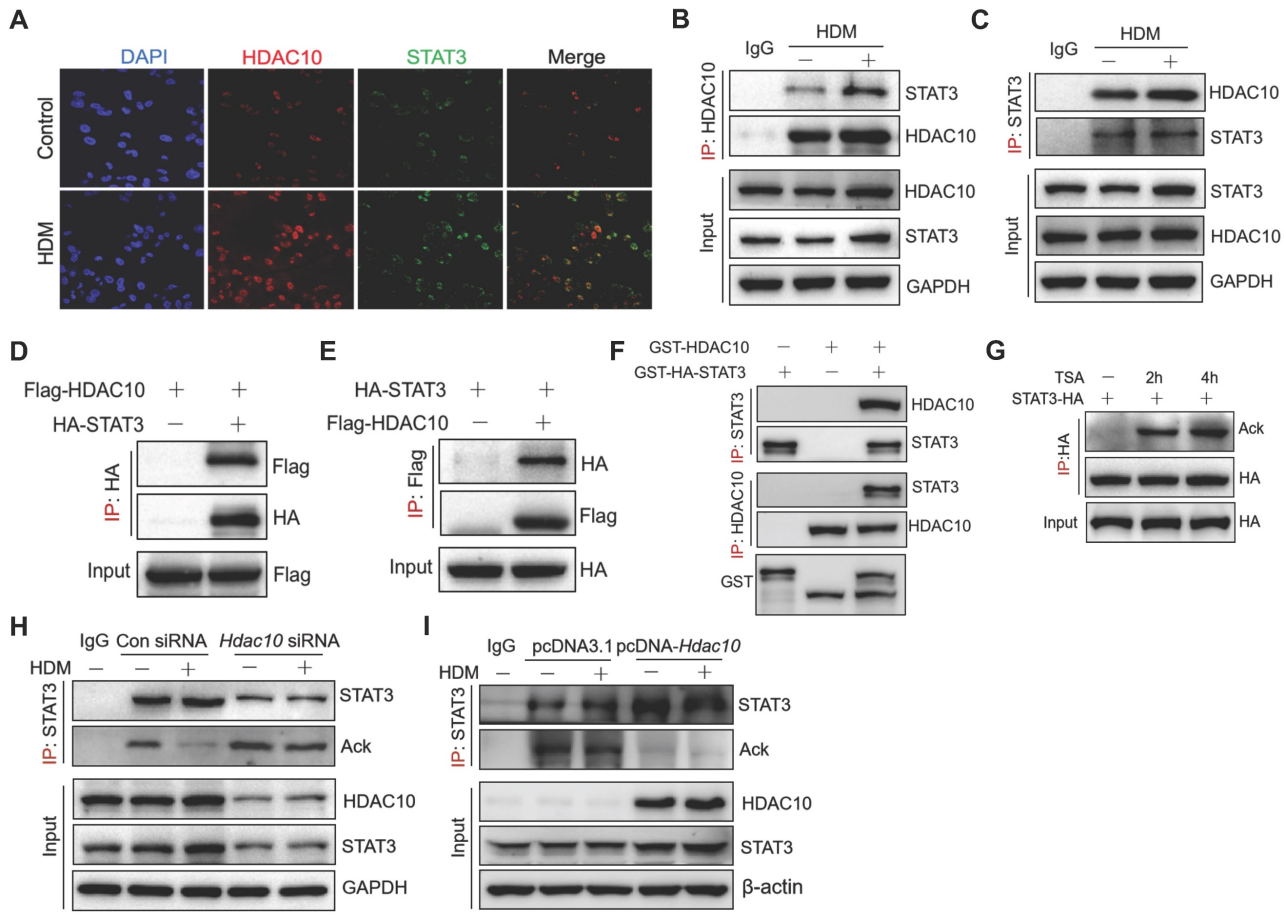
** HDAC10 directly interacted with STAT3 and targeted STAT3 for deacetylation. (A)** Confocal microscopy of the location of endogenous HDAC10 (Red) and STAT3 (Green) in THP1 cells treated with HDM for 24 h. DAPI, DNA binding dye. Images were captured at ×400 magnification. **(B, C)** Immunoblot (IB) analysis of endogenous HDAC10 or STAT3 in THP1 cells infected with HDM, assessed before (input) or after IP with IgG (control) or antibody to HDAC10 or STAT3. **(D, E)** IB analysis of exogenous HDAC10 or STAT3 in THP1 cells transfected with HA-tagged STAT3 alone or together with Flag-tagged HDAC10, assessed before (input) or after IP with antibody to HA or Flag. **(F)** IB analysis of HDAC10 and STAT3 interaction in a GST pull-down assay. **(G)** IB analysis of STAT3 acetylation (Ack) in THP1 cells transfected with HA-tagged STAT3 alone or together with histone deacetylase inhibitor Trichostatin A (TSA), assessed before (input) or after IP with antibody to STAT3 and Ack. **(H)** THP1 cells were transfected with Control siRNA or* Hdac10* siRNA for 24 h and then treated with HDM (100 μg/ml) for 24 h, and assessed before (input) or after IP with antibody to STAT3 and Ack. **(I)** THP1 cells were transfected with pcDNA3.1 or pcDNA-HDAC10 plasmid for 24 h and then treated with HDM (100 μg/ml) for 24 h, and assessed before (input) or after IP with antibody to STAT3 and Ack. Data are shown as means ± SEM. Data are representative of three independent experiments with similar results.

To investigate the mechanisms by which HDAC10 regulates STAT3 in asthma, we have therefore completed a series of experiments. Immunofluorescence assay indicated that HDAC10 and STAT3 were expressed in both the nucleus and cytoplasm, but mainly located in the nucleus of THP1 cells (Figure 7A). To determine whether HDAC10 and STAT3 physically interact, co-immunoprecipitation (Co-IP) analysis were performed. HDAC10 and STAT3 coimmunoprecipitated with each other and that allergen could up-regulate HDAC10 and STAT3 complex (Figure 7B-C). We further determined whether HDAC10 was able to interact with STAT3 exogenously. The exogenous HDAC10 and STAT3 complex was observed in THP1 cells (Figure 7D-E). Moreover, the direct interaction of HDAC10 and STAT3 was verified by glutathione S-transferase (GST) pull-down (Figure 7F).

Given that HDAC10 is a deacetylase, we investigated whether HDAC10 targets STAT3 for deacetylation. STAT3 was overexpressed in THP1 cells using HA-STAT3 plasmid and then treated with histone deacetylase inhibitor trichostatin A (TSA) for 2 h or 4 h. We immunoprecipitated HA-STAT3 with anti-HA and found that STAT3 was indeed acetylated, and its acetylation was enhanced after treatment with TSA (Figure 7G). To further illustrate the involvement of HDAC10 in STAT3 acetylation modification, *Hdac10* was knocked-down using a siRNA approach or was overexpressed using HDAC10 plasmid in THP-1 cells and then treated with allergen for 24 h. We found that HDM treatment decreased STAT3 acetylation in THP1 cells, but was inhibited by *Hdac10* knockdown using siRNA (Figure 7H). In contrast, HDAC10 overexpression in THP1 cells using HDAC10 plasmid significantly reduced STAT3 acetylation compared with the control cells exposed to allergen (Figure 7I). Collectively, this finding suggested that HDAC10 directly interacted with STAT3 and targeted STAT3 for deacetylation, which contributes to promote M2 program during allergic inflammation.

### HDAC10 inhibitor treatment attenuated allergic airway inflammation

Our data indicated that HDAC10 inhibition might represent a valuable therapeutic approach for asthma. Therefore, we identified a HDAC10 inhibitor using a molecular docking screening strategy as described in Methods. The docking scores of 5 molecules and HDAC10 structure and other basic information are shown in Table S3. To further screen compounds with good target inhibition activity, we used the protein structure of HDAC10 as the target for fine molecular docking with compound Salvianolic acid B (SAB). SAB, one of the main active ingredients of Salvia miltiorrhiza, has anti-inflammatory effects and a beneficial effect on cardiovascular diseases, cancers, and liver fibrosis 21-23. The interaction between HDAC10 and SAB were analyzed in detail. The interaction diagram of compound SAB with HDAC10 (Figure S4I) and the three-dimensional binding pattern diagram (Figure S4J) showed that the compound forms hydrogen bond interaction with Gly-145, Asp-174, Arg-198, Glu-276, Pro-273, Glu-274, suggesting that they are closely combined.

**Figure 8:**
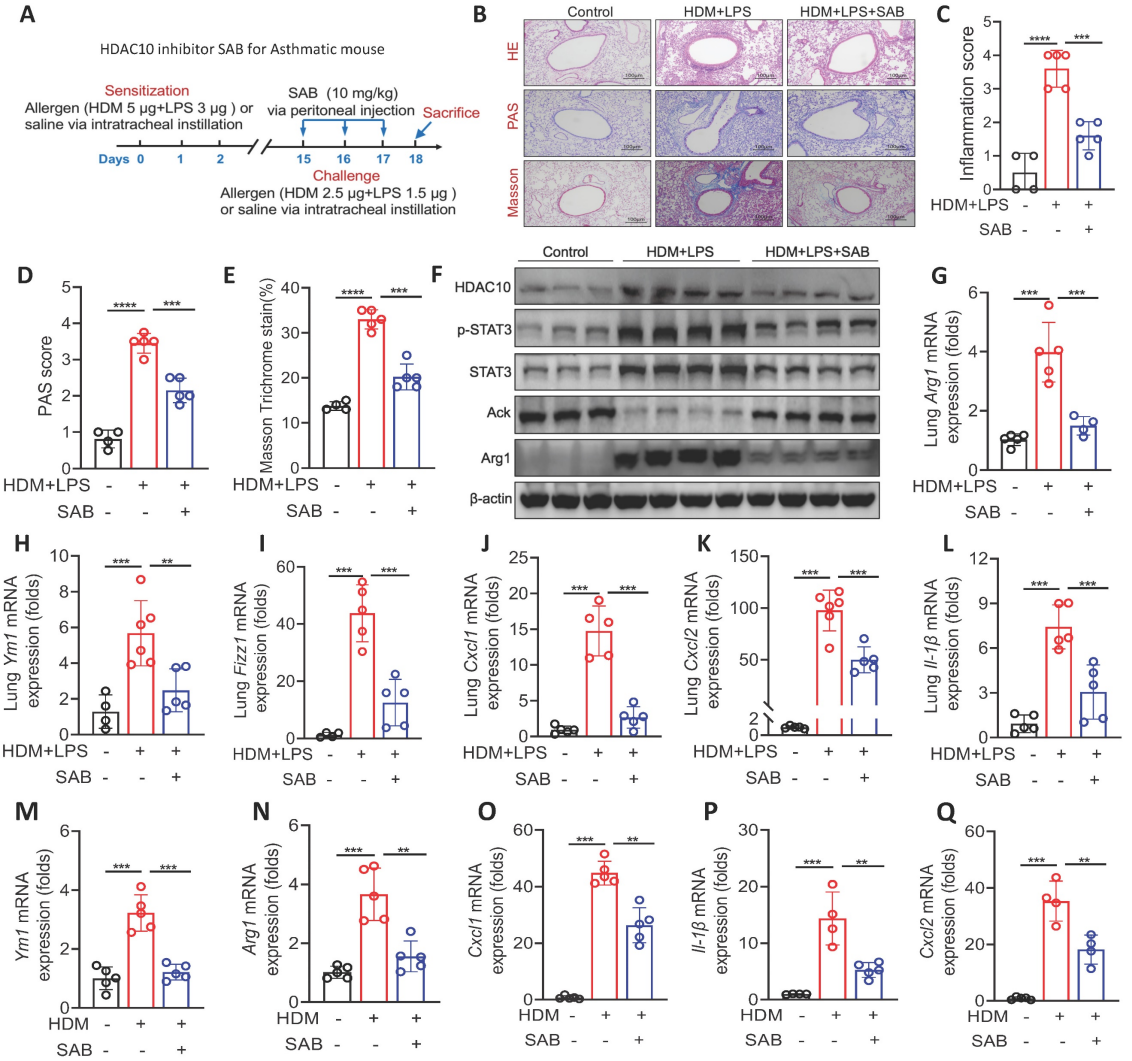
** The HDAC10 inhibitor Salvianolic acid B (SAB) prevented allergen-induced airway inflammation. (A)** Schematic overview of experimental design for **(B-Q)** in an asthma mouse model. **(B-E)** Representative photomicrographs of HE staining, PAS staining, and Masson staining in the lung tissue of mice are shown. Images were captured at ×200 magnification and quantification was done by using Image J software. **(F)** The expression of HDAC10, STAT3, and Arg1 in the lung homogenate of mice was analyzed by using Western blotting. **(G-I)** qRT-PCR analysis was conducted for macrophage M2 markers* Arg1*, *Fizz1*, and *Ym1* mRNA expression in the lung homogenate of mice. **(J-L)** The expression of inflammatory cytokines in the lung homogenate of mice was analyzed by using qRT-PCR. **(M, N)** The mRNA expression of *Arg1* and *Ym1* in BMDMs was conducted by qRT-PCR analysis. **(O-Q)** The levels of inflammatory cytokines in BMDMs was conducted by qRT-PCR analysis. Data are shown as means ± SEM (n = 4-6 mice/group). ^**^P < 0.01, ^***^P < 0.001, and ^****^P < 0.0001 versus Control (unless otherwise noted) by one-way ANOVA, followed by Tukey's multiple comparisons test. Data are representative of three independent experiments with similar results **(B and F)** or are from three independent experiments **(G-Q)**. See also Figure S4.

We further characterized the specificity of the HDAC10 inhibitor SAB in our study. BMDMs were isolated from WT mice and treated with HDM (100 μg/ml) and/or SAB (20 μM) for 24 h. The expression of HDACs (HDAC1-11) in BMDMs were analyzed using qRT-PCR. We found that the levels of* Hdac5*,* Hdac6*, *Hdac8*, *Hdac9*, and *Hdac10* were significantly increased in BMDMs treated with HDM compared with the control group, but only *Hdac10* was significantly decreased in BMDMs treated with HDM plus SAB (Figure S4K). We then evaluated the role of SAB in experimental mouse model as described in Figure 8A. HE staining, PAS staining, and Masson staining data demonstrated that SAB treatment attenuated allergen-induced airway inflammation, mucus secretion, and collagen deposition (Figure 8B-E). The expression of HDAC10, p-STAT3, STAT3, and Arg1 in the lung homogenates was obviously decreased by SAB administration compared with normal saline administration (Figure 8F, Figure S4L). SAB treatment also inhibited macrophage M2 markers (Arg1, Fizz1 and Ym1) and inflammatory cytokines in the lung homogenate of mice (Figure 8G-L). Consistent with animal data, SAB treatment inhibited macrophage M2 markers (Arg1, Fizz1, and Ym1) and inflammatory cytokines in HDM-induced BMDMs (Figure 8M-Q). Collectively, these findings suggested that inhibition of HDAC10 with SAB treatment may be of potential value in the treatment of airway inflammation in asthma.

## Discussion

In this study, we performed experiments in patients and animals to investigate the role of histone deacetylase HDAC10 on the pathogenesis of asthma. We found that HDAC10 expression was elevated in human biopsies, mouse models, and macrophages in response to allergen. Mice with a myeloid cell-specific *Hdac10* deficiency attenuated airway inflammation in asthmatic mice. We conjectured that HDAC10 directly interacts with STAT3 for its deacetylation in macrophages. The complex then promotes macrophage M2 polarization via PI3K/Akt signaling pathway. Importantly, we identified SAB as a HDAC10 inhibitor that effectively suppressed airway inflammation in asthmatic mice. Upregulation of HDAC10 was positively associated with disease severity of asthma. Collectively, our study disclosed a previously unknown HDAC10-STAT3 axis that facilitates macrophage M2 polarization and led to airway inflammation in asthma, implicating HDAC10 as a therapeutic target.

Epigenetic regulation plays a critical role in the effects of environmental factors in the pathogenesis of asthma 24. HDAC10 is a histone deacetylase that regulates melanogenesis in ovarian cancer patients 25. Oehme et al. demonstrated that HDAC10 protects cancer cells from cytotoxic agents 16. The expression of HDAC10 is inhibited in human renal cell carcinoma (RCC) cells, suggesting that HDAC10 is an independent predictor of the prognosis of RCC, and activating HDAC10 expression may be a new therapeutic strategy for advanced RCC 26. However, whether and how HDAC10 regulates the pathogenesis of asthma have not been reported. Here, we found that HDAC10 expression was elevated in asthmatic patients and mice. *Hdac10^fl/fl^*-*LysMCre* mice exhibited significantly reduced airway inflammation, cytokine production, and mucus production compared with *Hdac10^fl/fl^* mice exposed to allergens. Previous studies had shown that PI3K/Akt signaling participates in physiological functions such as regulating the expression of various inflammatory genes, DNA damage repair, and aging 18,19. PI3K/Akt signaling is also plays a role in macrophage M2 program 19. We found that the specific PI3K/Akt activator 1,3-DA significantly rescued the reduced airway inflammation and macrophage M2 program in *Hdac10^fl/fl^*-*LysMCre* mice after allergen stimulation. Specifically, the macrophage depletion and adoptive transfer experiments confirmed that reduced M2 macrophages protected against allergen-induced airway inflammation. Moreover, we identified SAB as a HDAC10 inhibitor that effectively suppressed airway inflammation in asthmatic mice.

STAT3 plays a critical role in determinant of polarization of the alternatively activated M2 macrophages 20. In our study, STAT3 expression was decreased in *Hdac10* deficient BMDMs. Consistent with *in vitro* experiments, STAT3 expression was significantly reduced in M2 macrophages of *Hdac10^fl/fl^*-*LysMCre* mice compared with *Hdac10^fl/fl^* mice exposed to allergen. Furthermore, compared with the control treatment, STAT3 selective agonist Colivelin treatment increased airway inflammation, lung inflammatory cytokines, and macrophage M2 markers (Arg1, Fizz1, and Ym1) in *Hdac10^fl/fl^*-*LysMCre* mice exposed to allergen. These results indicated that* Hdac10* deficiency suppressed STAT3 to attenuate macrophage M2 program during allergic inflammation. However, the mechanism of HDM in observed intracellular signaling mechanisms like HDAC10/STAT3 expression and activation remains unknown and needs further investigation.

Acetylation plays a critical regulatory role in protein-protein interactions. Acetylation of histones causes the relaxation of chromatin structure as a necessary but not sufficient condition for gene transcription 27. Deacetylation is also linked directly to gene transcription. H4K16 deacetylation is required for regulating transcription activation and promotes regional gene expression 28. STAT3 is involved in the pathology of asthma 20. However, the regulatory mechanisms remain unclear. To the best of our current knowledge, no studies have addressed whether and how HDAC10 regulates STAT3 expression and promotes macrophage M2 program in asthma. We discovered that HDAC10 directly interacted with STAT3 and targeted STAT3 for deacetylation. The complex was then promoted macrophage M2 polarization via PI3K/Akt signaling pathway. However, the more detailed mechanisms of STAT3 deacetylation that regulates STAT3 transcriptional activity still need to be further studied.

The present study does have some limitations. First, the *Hdac10^fl/fl^-LysMCre* mice were conditionally knocked out for HDAC10 in macrophages (CKO). HDAC10 is expressed in various types of cells, such as epithelial cells, endothelial cells and T cells 29,30. Further experiments are needed to investigate the effect of other cells on HDAC10 in the regulation of BMDMs and the role of HDAC10 in other cells. Second, although SAB has the ability to inhibit HDAC10 activity and transcription, the precise mechanisms underlying the impact of SAB on HDAC10 expression and activity remain elusive and warrant further investigation. Therefore, caution should be exercised when interpreting the role of SAB in asthma due to this limitation.

In conclusion, we have shown that HDAC10 was highly expressed in macrophages and *Hdac10* deficiency protected mice against allergen-induced airway inflammation via PI3K/Akt signaling. We conjectured that HDAC10 directly interacts with STAT3 and targeted STAT3 for deacetylation to enhance an M2 program. Moreover, HDAC10 inhibitor treatment attenuated allergic airway inflammation. These data provided insight into the relation of epigenetic regulators with STAT3 in macrophage M2 program and supplied important clues for a potential strategy against airway inflammation in asthma.

## Materials and methods

### Human samples

The diagnosis of asthma was based on the Global Initiative on Asthma (GINA) guidelines 31. Asthmatic patients between 18 and 65 years old were included. Exclusion criteria were combined respiratory diseases other than asthma such as chronic obstructive pulmonary disease (COPD), and lung cancer. Patients who had lung nodules and underwent surgery were used as normal control. Bronchial biopsies were obtained from patients undergoing bronchoscopy for diagnostic purposes. Peripheral blood mononuclear cells (PBMC) were isolated from peripheral blood samples of study subjects using density gradient centrifugation according to manufacturer instructions. Clinical information is summarized in Table S1 and S2. The study protocols were approved by the Medical Ethics Committee of Affiliated Hospital of Guangdong Medical University (PJKT2022-079). Written informed consent was obtained from all participants.

### Animal studies

*Hdac10^fl/fl^* mice were generated using the CRISPR-Cas9 system by the Cyagen Biosciences Inc. (Suzhou, China). Two loxP sequences were inserted in the introns flanked with the exon 2 and exon 14 of HDAC10 as described in Figure 2A. The *LysMCre* mice were a generous gift from Dr. G. Feng (University of California at San Diego, CA, USA). *Hdac10^fl/fl^*-*LysMCre* mice were generated by crossing the *LysMCre* mice with *Hdac10^fl/fl^* mice for specific deletion of *Hdac10* in macrophages. All experimental procedures were approved by the Guangdong Medical University's Animal Ethical Committee.

### Allergic asthma mouse model

Allergic asthma mouse model was established according to we and others previously described 32-34. Briefly, six to eight-week-old mice were sensitized by intratracheal instillation of 5 μg HDM plus 3 μg LPS in 50 μl normal saline (NS) on days 0, 1, and 2. On days 15, 16 and 17 after the initial sensitization, the mice were challenged with 2.5 μg HDM plus 1.5 μg LPS in 50 μl NS using intratracheal instillation. Mice were administered with same volume of normal saline served as controls. The HDAC10 inhibitor (Salvianolic acid B, SAB), specific PI3K/Akt activator 1,3-Dicaffeoylquinic acid (DA) or STAT3 agonist (Colivelin) was injected intraperitoneally into the mice 2 h before the 2.5 μg HDM plus 1.5 μg LPS intratracheal instillation. The mice were sacrificed under anesthesia 24 h after the final challenge.

### Histological and immunohistochemical analysis

Lung tissue sections were subjected to hematoxylin and eosin (HE) staining, Masson's trichrome staining (Masson), and Periodic Acid-Schiff (PAS) staining. For immunostaining, the frozen sections were incubated with antibodies against CD206, STAT3, HDAC10, and F4/80, followed by staining with Alexa Fluor 594-labeled anti-mouse/rabbit or Alexa Fluor 488-conjugated anti-rabbit/mouse antibodies, respectively. All slides were examined in a random blinded fashion by two independent investigators. At least 10 bronchioles were counted on each slide and the data used for statistical analysis.

### Quantitative RT-PCR (qRT-PCR)

Total RNA was isolated from the cells and mice lung tissues using Trizol reagent (Invitrogen, Carlsbad, CA, USA) and was reverse-transcribed into cDNA using the PrimeScript™RT reagent kit (Takara, Beijing, China). cDNA was used for RT-PCR amplification using TB Green™ Premix Ex Taq™ (Takara, Beijing, China). The expression of individual genes was normalized to the expression of β-actin. The primers used to amplify each target gene were shown in Table S4.

### Culture and treatment of BMDMs

BMDMs were isolated and cultured as we previously reported 7. Briefly, BMDMs were collected from six-to eight-week-old male mice. Red blood cells (RBCs) Lysing Buffer (Tbdscience.com) was used to remove red blood cells. The remaining cells were incubated in DMEM containing antibiotics, 10% fetal bovine serum (FBS), and 10 ng/ml recombinant mouse M-CSF (Novoprotein, Catalog #0331488) for 7 days to promote differentiate bone marrow-derived macrophages. After 7 days, the differentiated macrophages were cocultured with IL-4 (10 ng/ml) or HDM (0, 25, 50, 75, 100 μg/ml) at the indicated time points.

### Dual-luciferase reporter assay

*Hdac10^fl/fl^* and *Hdac10^fl/fl^-LysMCre* BMDMs transfected with Renilla TK luciferase reporter vectors, luciferase reporter vectors and pSTAT3-TA-luc plasmid for 24 h, and then treated with HDM (100 μg/ml) for another 24 h. The cells were collected for luciferase activity assays using the Dual-Luciferase Reporter Assay System kit (Promega) according to the manufacturer's instructions.

### Macrophage depletion and adoptive transfer studies

According to previous study 18, WT mice were injected with Clodronate liposomes (200 μl) or equal volume liposomes through the caudal vein for two consecutive days. Mice were sacrificed 24 h after intravenous injection to evaluate the macrophage depletion efficiency. For adoptive transfer studies, the *Hdac10^fl/fl^* and *Hdac10^fl/fl^*-*LysMCre* naive BMDMs were first stimulated with IL-4 (10 ng/ml) or equal volume of NS for 12 h to induce M2 macrophage polarization, and then transferred into the lungs of liposome-treated WT mice at a density of 1×10^6^ cells per mouse (50 μl) on days 16 and 17 of allergen induction as described above through intratracheal instillation. The mice were sacrificed 24 h after adoptive transplantation for analysis.

### Western blotting

Total proteins were extracted from lung tissues and cells using RIPA, and the concentration was detected by BCA protein assay kit according to the manufacturer's protocol (Beyotime, Shanghai, China). Equal amounts of protein were separated via New Semet Express-cast PAGE color gel electrophoresis and electrophoresed onto PVDF membranes. The membranes were blocked with 5% skim milk or 5% BSA for 1 h, and incubated with primary antibodies incubated at 4 °C overnight. After washed with 1×TBST solution for 3 times, the PVDF membrane was incubated with the corresponding secondary antibody for 1 h. Then the PVDF membrane was washed with 1×TBST solution 3 times again, and the exposure was analyzed and sorted out.

### Co-immunoprecipitation (Co-IP)

After Total proteins of cells were extracted with cell lysis buffer, the protein concentration was determined by the BCA method and 1mg protein was taken for subsequent immunoprecipitation. Each sample was incubated with 1 µg antibody overnight at 4 °C, then incubation with 30 μl of Protein G Beads at 4 °C for 2 h. The immunocomplexes were washed 5 times with IP lysis buffer, then resuspended with 2×SDS Sample Buffer for Western blotting.

### GST pull-down

GST-HDAC10 and GST-HA-STAT3 plasmids were constructed. The GST fusion protein was expressed in E. coli BL21 cells and purified according to manufacturer's instructions using the GST-Tag Protein Purification Kit (Beyotime). GST-HDAC10 and GST-HA-STAT3 proteins were mixed in equal amounts using the BCA kit to quantify the protein concentration, and then the corresponding primary antibody was added and incubated at 4 °C overnight. On the second day, each sample was added with the same amount of Protein G Beads and incubated at 4 °C for 1~2 h. After washing, western blotting was performed with anti-HDAC10 or anti-STAT3 antibody.

### ELISA

Mouse CXCL1 and mouse CXCL2 in BALF and lung homogenate of mice were measured using ELISA kit (Elabscience). Assays were according to the manufacturer's protocol.

### Flow cytometry

The cultured naïve BMDMs were stimulated by IL-4 (10 ng/ml) for 12 h. The cells were stained with anti-mouse CD206-PerCP/Cy5.5 and F4/80-APC. All staining processes were performed according to recommended protocols. The staining was detected by flow cytometry (BD FACSCantoⅡ) as previously described 35, and the data were analyzed using Flow Jo v10 (Becton Dickinson).

### Molecular docking

The molecular docking program consists of two parts. First, in order to rapidly screen out molecules with good target binding activity, we first combined the traditional Chinese medicine database with HDAC10 (PDB; 5TD7) to perform rapid virtual filtering. In the next step, the compound Salvianolic acid B with the highest scoring data was matched with the target carefully, and the sites were selected as Glu 272, Asp 177 and Gln 302. Finally, we show the docking scoring table and the combination model diagram 36,37.

### Reagents and antibodies

Please see the Table S5.

### Statistical analysis

Unless stated otherwise, data are presented as means ± SEM. The data were analyzed using GraphPad Prism (San Diego, CA, version 8.0). Two experimental groups were compared using Mann-Whitney U-test or Student's *t* test. For comparisons between more than two groups, an ordinary one-way analysis of variance (ANOVA) followed by Tukey's post hoc test. Categorical variables were tested using Chi-square. A *P* value of < 0.05 was considered statistically significant.

## Supplementary Material

Supplementary tables.

Supplementary figures.

## Abbreviations

HDAC10: Atopic dermatitis
STAT3: Expression quantitative trait locus
BMDMs: Formaldehyde-assisted isolation of regulatory elements
SAB: Salvianolic acid B
DA: 1,3-Dicaffeoylquinic acid
HDM: house dust mites
LPS: lipopolysaccharide
PBMCs: peripheral blood mononuclear cells
