# Supplementary material for: The HDAC10 instructs macrophage M2 program via deacetylation of STAT3 and promotes allergic airway inflammation
Source: Theranostics. 2023 Jun 19;13(11):3568–81. doi: 10.7150/thno.82535 (PMC10334828; doi:10.7150/thno.82535)
Supplement: Supplementary file 1 — Supplementary tables. [file thnov13p3568s1.pdf]

Table S1. ***Characteristics of the subjects for bronchial biopsies study***

Groups	Age (ys)	Sex	HDAC10 (%)*	p-Akt (%)*	p-P85 (%)*	Eos (%)	Clinical diagnosis
Control	48	M	11.5	14.6	12.3	2.11	Lung nodules
	51	M	12.3	16.1	12.5	3.27	Lung nodules
	55	M	14.5	14.2	14.1	1.78	Lung nodules
	60	M	10.8	15.9	16.1	1.86	Lung nodules
	57	F	15.3	14.1	13.9	2.29	Lung nodules
Asthmatic patients	51	F	35.6	55.2	35.6	5.56	Mild asthma
	58	M	43.5	48.3	38.7	6.23	Mild asthma
	50	F	55.3	54.6	44.9	5.28	Moderate asthma
	47	F	48.3	48.5	49.1	5.27	Moderate asthma
	32	F	37.9	37.9	37.3	6.08	Moderate asthma
	65	M	42.6	46.2	36.9	10.25	Severe asthma
	48	F	38.6	39.7	30.2	8.26	Severe asthma
	66	F	29.4	48.3	47.6	7.23	Severe asthma
	48	M	36.5	41.2	41.9	11.59	Severe asthma

* Positive cells in airway biopsy (% of total cells)

Table S2. ***Characteristics of the subjects for peripheral blood study***

Parameters	Control (n = 8)	Asthma (n = 8)	P value
**Age, yrs**	28.0 ± 1.36	31.1 ± 2.2	0.248
**Male/Female**	3/5	4/4	0.614
**FEV_1, L**	3.00 ± 0.17	1.75 ± 0.28	0.002
**FVC, L**	3.53 ± 0.20	2.66 ± 0.32	0.040
**FEV_1/FVC, %**	101.90 ± 2.99	73.27 ± 4.46	0.001
**ACT score**	NA	19.13± 0.97	NA

Data are presented as mean ± SEM, unless otherwise stated.

FEV_1, forced expiratory volume in 1 second; FVC, forced vital capacity; ACT, asthma control test; N/A, not applicable.

Table S3. Molecular docking results of 5 selected ligands from traditional Chinese medicine (TCM) monomer database

Molecules	2D Structure	Binding Affinity (kcal/mol)	Formula	CID
Salvianolic acid B	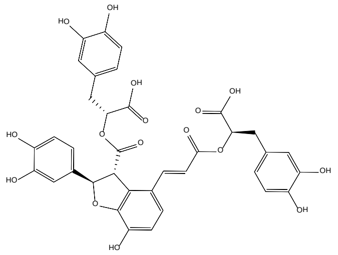	-8.90	C36H30O16	11629084
Isoacteoside	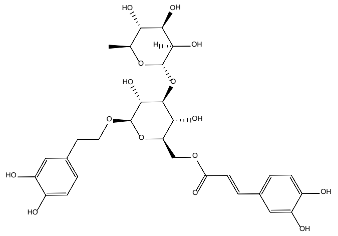	-8.71	C29H36O15	6476333
Angoroside C	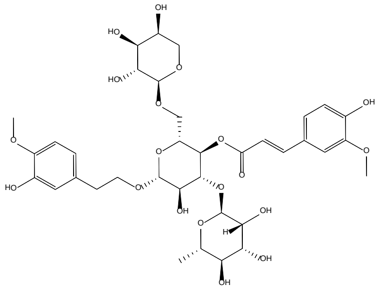	-8.7	C36H48O19	23757181
Lithospermic acid B	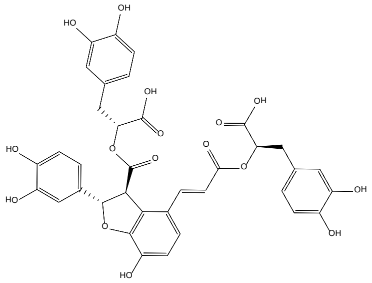	-8.6	C36H30O16	6451084
oxypropanoic acid	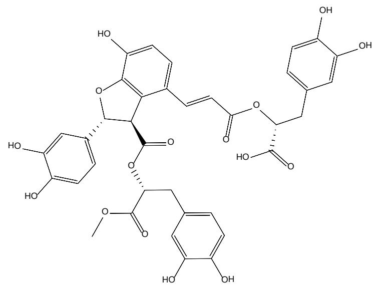	-8.51	C37H32O16	122169312
Panobinostat	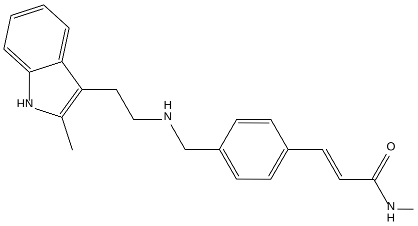	-8.51	C19H14N2O6$_7$	6918837

Table S4. Primers used for quantitative real time PCR analysis

Species/genes	Primer sequence
m-Hdac10 forward	5′-ACAGCCACTCGACTGCTCT-3′
m-Hdac10 reverse	5′- GATGCCTCACAAGCTGACAAA -3′
m-Cxcl-1forward	5′-CTGGGATTCACCTCAAGAACATC-3′
m-Cxcl-1 reverse	5′-CAGGGTCAAGGCAAGCCTC-3′
m-Cxcl-2 forward	5′-TGTCCCTCAACGGAAGAACC-3′
m-Cxcl-2 reverse	5′-CTCAGACAGCGAGGCACATC-3′
m- Il-1 β forward	5′-GCAACTGTTCCTGAACTCAACT-3′
m- Il-1 β reverse	5′-ATCTTTTGGGGTCCGTCAACT -3′
m-Arg1 forward	5′-CTGACCTATGTGTCATTTGG-3′
m-Arg1 reverse	5′-CATCTGGGAACTTTCCTTTC-3′
m-Ym1 forward	5′-GGGCATACCTTTATCCTGAG-3′
m-Ym1 reverse	5′-CCACTGAAGTCATCCATGTC-3′
m-Fizz1 forward	5′-TCCCAG TGAATA CTG ATGAGA-3′
m-Fizz1 reverse	5′-CCACTCTGGATCTCCCAAGA-3′
m-actin forward	5′-AGTGTGACGTTGACATCCGT-3′
m-actin reverse	5′-GCAGCTCAGTAACAGTCCGC-3′

m: mouse

Table S5 Key resources

REAGENT or RESOURCE	SOURCE	IDENTIFIER
**Antibodies**		
Anti-β-actin	Beyotime	Cat#AA128
Anti-Tubulin	Beyotime	Cat#AF0001
Anti-GAPDH	Affinity	Cat#AF7021
Anti-HDAC10	Santa Cruz	Cat#sc-393417
Anti-STAT3	Santa Cruz	Cat#E1722
Anti-GST	Santa Cruz	Cat#sc-138
Anti-HA	Abbkine	Cat#ABT2040
Anti-Acetyl Lysine	Immunechem	Cat#ICP0380
Alexa Fluor 488	Beyotime	Cat#A0423
Alexa Flour 555	Beyotime	Cat#A0460
Anti-F4/80	Proteintech	Cat#29414-1-AP
Anti-CD206	Proteintech	Cat#11265-1-AP
Clophosome-A-Clodronate Liposomes	FormuMax	Cat#F70101C-A-2
Anti-F4/80 (APC)	Biolegend	Cat#121116
Anti-CD206 (Percp-Cy5.5)	Biolegend	Cat#141716
Anti p-P85	Beyotime	Cat#AF5905
Anti P85	Proteintech	Cat#60025-1-1g
Anti p-Akt	Proteintech	Cat#28731-1-AP
Anti Akt	Beyotime	Cat#AF0045
Anti ARG1	Beyotime	Cat#AF1381
**Chemicals**		
Salvianolic acid B (SAB)	SparkJade	Cat#SJ-MN0081
Colivelin	SparkJade	Cat#SJ-BP0032
1,3-Dicaffeoylquinic acid (1,3-DA)	https://www.bjbal.com	Cat#M05856
HDM	GREER	Cat#XPB82D3A25
LPS	SIGMA	Cat#L2880-25MG
TSA	Beyotime	Cat#P1112
DAPI	Beyotime	Cat#C1005
Recombinant Murine IL-4	Beyotime	Cat#P5916-5μg
Protein A+G Agarose	Beyotime	Cat#P2055-50ml
Recombinant mouse M-CSF	Novoprotein	Cat#CB34

To be continued

**Critical commercial assays**		
GST-tag Protein Purification Kit	Beyotime	Cat#P2262
Mouse GROα/CXCL1 ELISA Kit	Elabscience	Cat#E-EL-M0018c
Mouse GROβ/CXCL2 ELISA Kit	Elabscience	Cat#E-EL-M0019c
TB Green® Premix Ex Taq™	Takara	Cat#RR420A
Prime Script™ RT reagent Kit	Takara	Cat#RR047A
Express Cast PAGE Gel Preparation kit	NCM Biotech	Cat#P2012
**Plasmid**		
pcDNA3.1-HA	Yubo Biotechnology, China	
HA-STAT3	Yubo Biotechnology, China	
pSTAT3-TA-luc	Yubo Biotechnology, China	
GST-HA-STAT3	Yubo Biotechnology, China	
GST-HDAC10	Yubo Biotechnology, China	
**Software and Algorithms**		
GraphPad Prism 8.0	https://www.graphpad.com	N/A
Image J	https://imagej.net/Welcome	N/A
Real-Time PCR Systems	Applied Biosystem	N/A
**Mice**		
C57BL/6 wild-type (WT) mice	GemPharmatech Co., Ltd., China	N/A
Hdac10$^{fl/fl}$ mice	GemPharmatech Co., Ltd., China	N/A
