# Supplementary material for: The HDAC10 instructs macrophage M2 program via deacetylation of STAT3 and promotes allergic airway inflammation
Source: Theranostics. 2023 Jun 19;13(11):3568–81. doi: 10.7150/thno.82535 (PMC10334828; doi:10.7150/thno.82535)
Supplement: Supplementary file 2 — Supplementary figures. [file thnov13p3568s2.pdf]

Supplemental figure legends

Figure S1. HDAC10 expression was increased in THP1 cells and BMDMs exposed to allergen, related to Figure 1

(**A**) THP1 cells were stimulated with HDM (100 μg/ml) for 24 h and representative immunofluorescence (IF) staining of HDAC10 is shown. Images were captured at ×400 magnification.

(**B, C**) Western blotting analysis of HDAC10 in allergen-induced THP1 cells for different doses or times. Quantification was done by using Image J software.

(**D**) BMDMs were stimulated with HDM (100 μg/ml) for 24 h and representative IF staining of HDAC10 is shown. Images were captured at ×400 magnification.

(**E, F**) Western blotting analysis of HDAC10 in allergen-induced BMDMs for different doses or times. Quantification was done by using Image J software.

Data are shown as means ± SEM. $^{*}P < 0.05$, $^{**}P < 0.01$, and $^{***}P < 0.001$ versus Control (unless otherwise noted) by one-way ANOVA, followed by Tukey's multiple comparisons test. Data are representative of three independent experiments with similar results (A-C and D-F).

Figure S2. *Hdac10* deficiency repressed inflammatory cytokine production after allergen induction, related to Figure 2

(**A-D**) qRT-PCR and ELISA analysis were conducted for inflammatory cytokines in the lung homogenate from $Hdac10^{fl/fl}$ and $Hdac10^{fl/fl}$-*LysMCre* mice after allergen exposure.

(**E, F**) ELISA analysis was conducted for inflammatory cytokines in the bronchoalveolar lavage fluid (BALF) from $Hdac10^{fl/fl}$ and $Hdac10^{fl/fl}$-*LysMCre* mice after allergen induction.

(**G, H**) BMDMs were isolated from $Hdac10^{fl/fl}$ and $Hdac10^{fl/fl}$-*LysMCre* mice and stimulated in vitro with HDM for 24 h. Inflammatory cytokines were assessed by qRT-PCR analysis.

Data are shown as means ± SEM. $^{**}P < 0.01$ and $^{***}P < 0.001$ versus Control (unless otherwise noted) by one way ANOVA, followed by Tukey's multiple comparisons test.

Data are from three independent experiments.

Figure S3. *Hdac10* deficiency attenuated the macrophage M2 program, related to Figure 3

(**A, B**) Clodronate liposomes were intratracheally administered to deplete macrophages in the lungs of WT mice as described in Methods. Representative IF staining of F4/80 in the lung sections of mice after depletion of macrophages is shown. Images were captured at ×200 magnification and quantification was done by using Image J software.

(**C-J**) The details of adoptive transfer studies as described in Methods. Macrophage M2 marker levels (*Arg1* and *Ym1*), HE staining, inflammatory cytokines in the lung tissue were increased in allergen-challenged WT mice with depletion of macrophages compared with controls after transfer of $Hdac10^{fl/fl}$ BMDMs. On the contrary, the Macrophage M2 marker levels were decreased in allergen-challenged WT mice with depletion of macrophages compared with controls after transfer of $Hdac10^{fl/fl}$-*LysMCre* BMDMs.

Data are shown as means ± SEM. $^{*}P < 0.05$, $^{**}P < 0.01$, $^{***}P < 0.001$, and $^{****}P < 0.0001$ versus Control (unless otherwise noted) by two-tailed unpaired Student's t test or one-way ANOVA, followed by Tukey's multiple comparisons test. Data are representative of three independent experiments with similar results (A and E) or are from three independent experiments (C-D, and G-I).

Figure S4. Supplementary information for Figure 4, 6, and 8.

(**A-D**) Quantification of Western blotting was done by using Image J software. Related to Figure 4G.

(**E**) Schematic overview of experimental design for PI3K/Akt activator 1,3-DA for asthmatic mouse. Related to Figure 4.

(**F-H**) Quantification of Western blotting was done by using Image J software. Related to Figure 6B.

(**I**) Analysis of interaction forces between compound Salvianolic acid B (SAB) and HDAC10. Related to Figure 8.

(**J**) Binding pattern analysis of compound SAB and HDAC10. Related to Figure 8.

(K) BMDMs were isolated from WT mice and treated with HDM (100 μg/ml) or HDM (100 μg/ml) plus SAB (20 μM) for 24 h. The expression of HDACs (HDAC1-11) in BMDMs were analyzed using qRT-PCR. Related to Figure 8.

(L) Quantification of Western blotting was done by using Image J software. Related to Figure 8F.

Figure S5. Graphical Abstract

Proposed working model of the function of HDAC10 instructs macrophage M2 program via deacetylation of STAT3 and promotes allergic airway inflammation.

Fig. S1

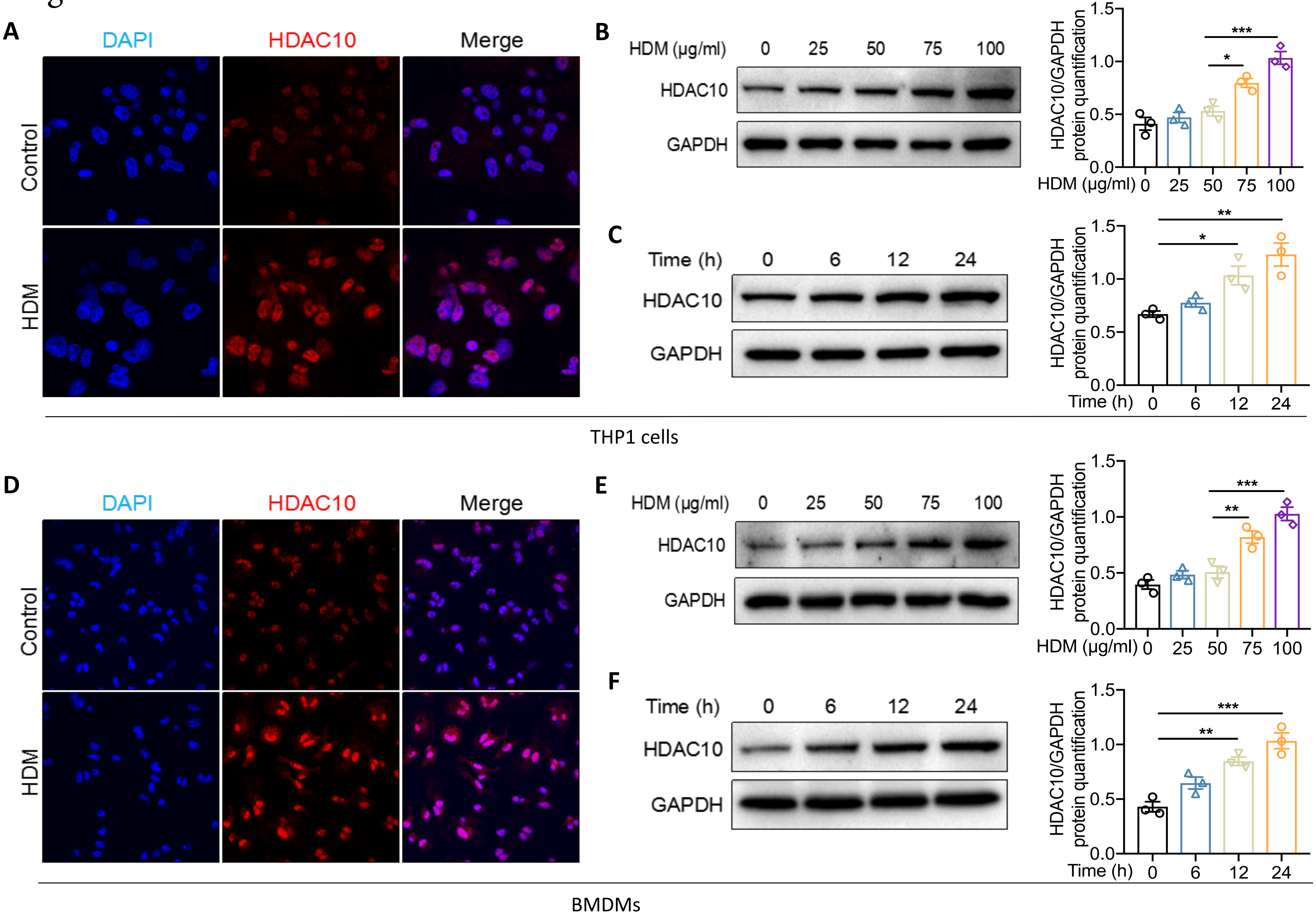

A
DAPI
HDAC10
Merge
Control
HDM
B
HDM (μg/ml) 0 25 50 75 100
HDAC10
GAPDH
HDAC10/GAPDH protein quantification
HDM (μg/ml) 0 25 50 75 100
C
Time (h) 0 6 12 24
HDAC10
GAPDH
HDAC10/GAPDH protein quantification
Time (h) 0 6 12 24
THP1 cells
D
DAPI
HDAC10
Merge
Control
HDM
E
HDM (μg/ml) 0 25 50 75 100
HDAC10
GAPDH
HDAC10/GAPDH protein quantification
HDM (μg/ml) 0 25 50 75 100
F
Time (h) 0 6 12 24
HDAC10
GAPDH
HDAC10/GAPDH protein quantification
Time (h) 0 6 12 24
BMDMs

Fig. S2

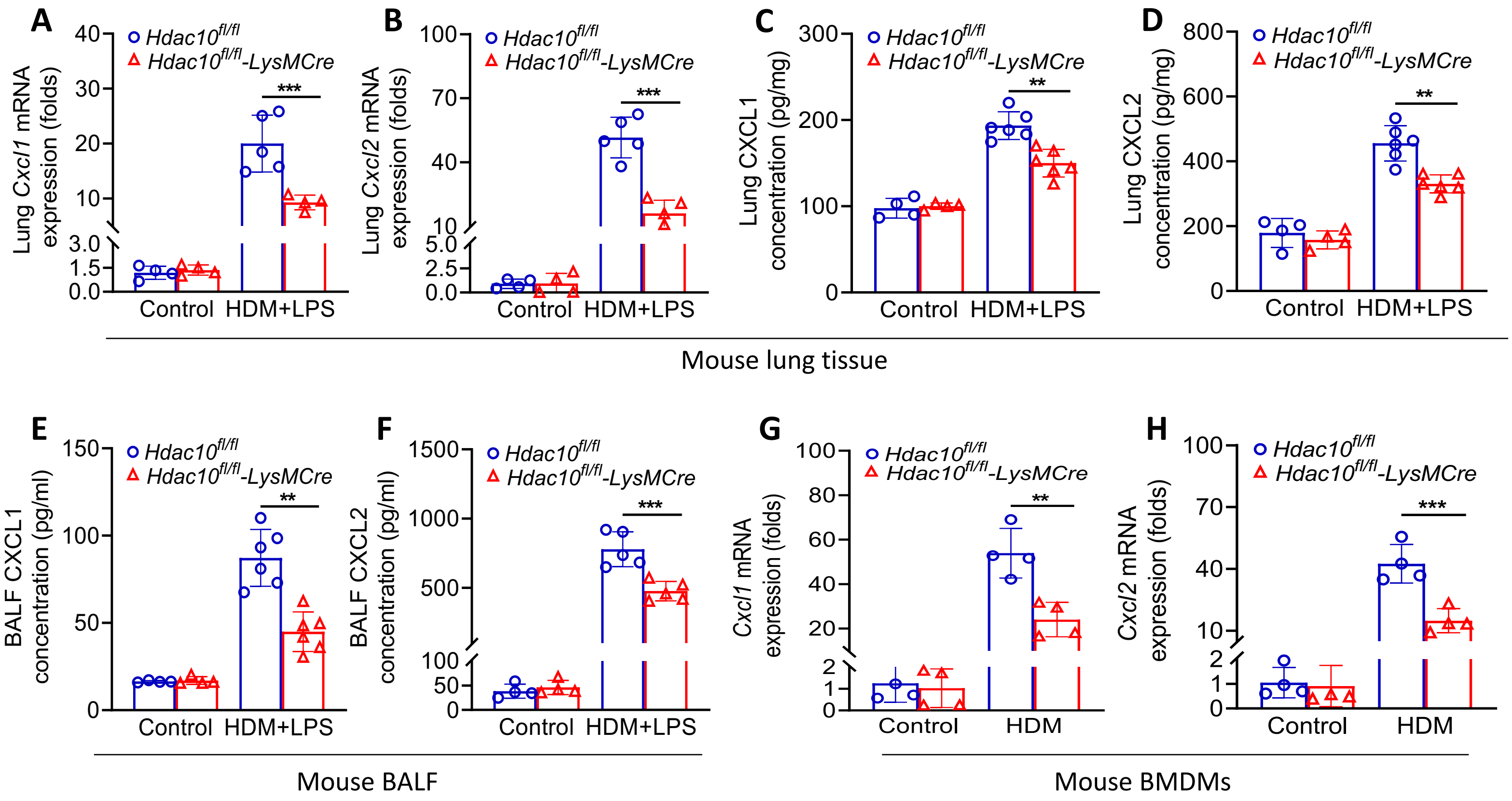
A
B
C
D
E
F
G
H
Hdac10fl/fl
Hdac10fl/fl-LysMCre
Lung Cxcl1 mRNA expression (folds)
Lung Cxcl2 mRNA expression (folds)
Lung CXCL1 concentration (pg/mg)
Lung CXCL2 concentration (pg/mg)
BALF CXCL1 concentration (pg/ml)
BALF CXCL2 concentration (pg/ml)
Cxcl1 mRNA expression (folds)
Cxcl2 mRNA expression (folds)
Control
HDM+LPS
HDM
***
**
Mouse lung tissue
Mouse BALF
Mouse BMDMs

Fig. S3

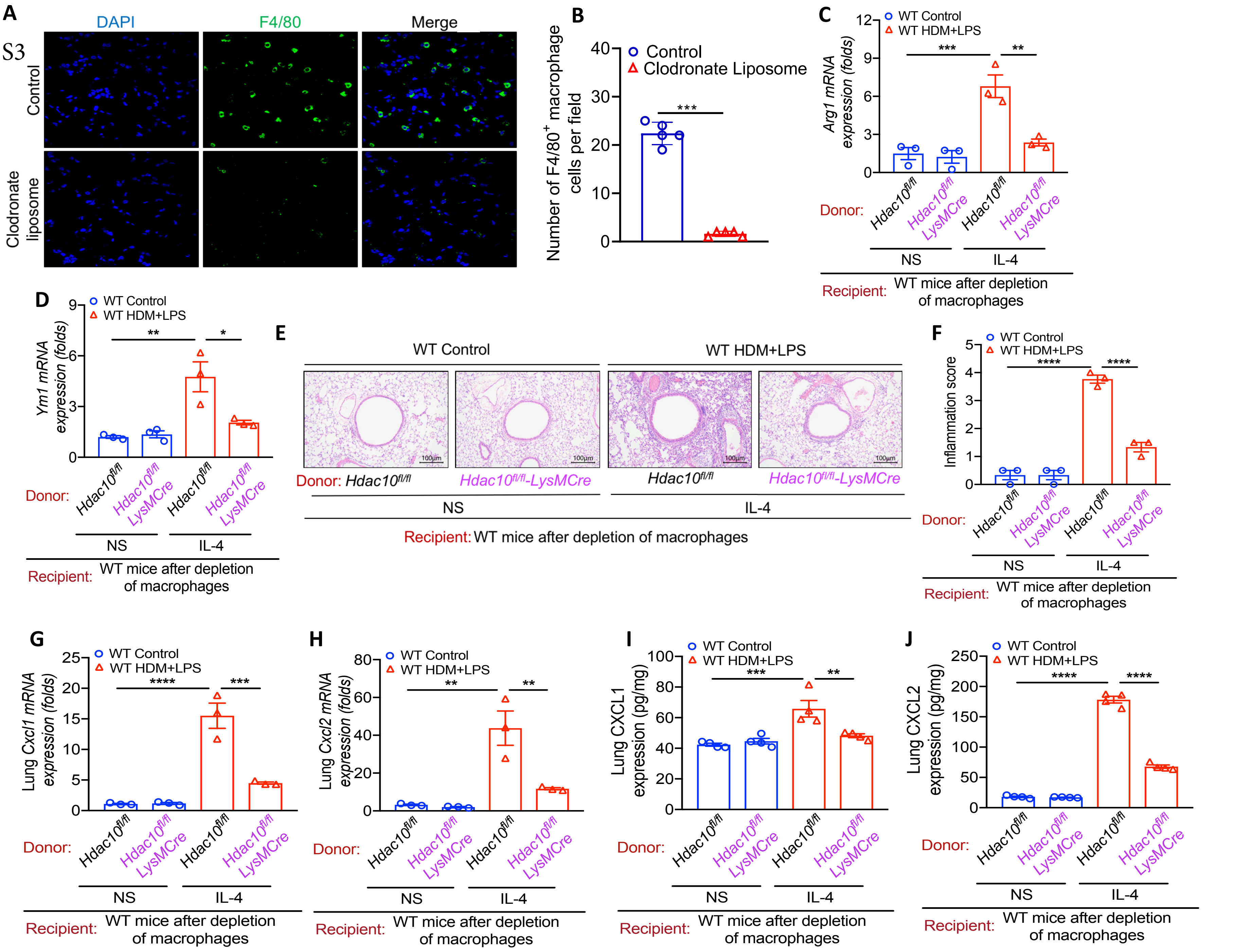
A
DAPI
F4/80
Merge
Control
Clodronate liposome
B
Control
Clodronate Liposome
Number of F4/80+ macrophage cells per field
C
WT Control
WT HDM+LPS
Arg1 mRNA expression (folds)
Donor:
Hdac10fl/fl
Hdac10fl/fl LysMCre
NS
IL-4
Recipient:
WT mice after depletion of macrophages
D
Ym1 mRNA expression (folds)
E
WT Control
WT HDM+LPS
Donor: Hdac10fl/fl
Hdac10fl/fl-LysMCre
Recipient: WT mice after depletion of macrophages
F
Inflammation score
G
Lung Cxcl1 mRNA expression (folds)
H
Lung Cxcl2 mRNA expression (folds)
I
Lung CXCL1 expression (pg/mg)
J
Lung CXCL2 expression (pg/mg)

Fig. S4

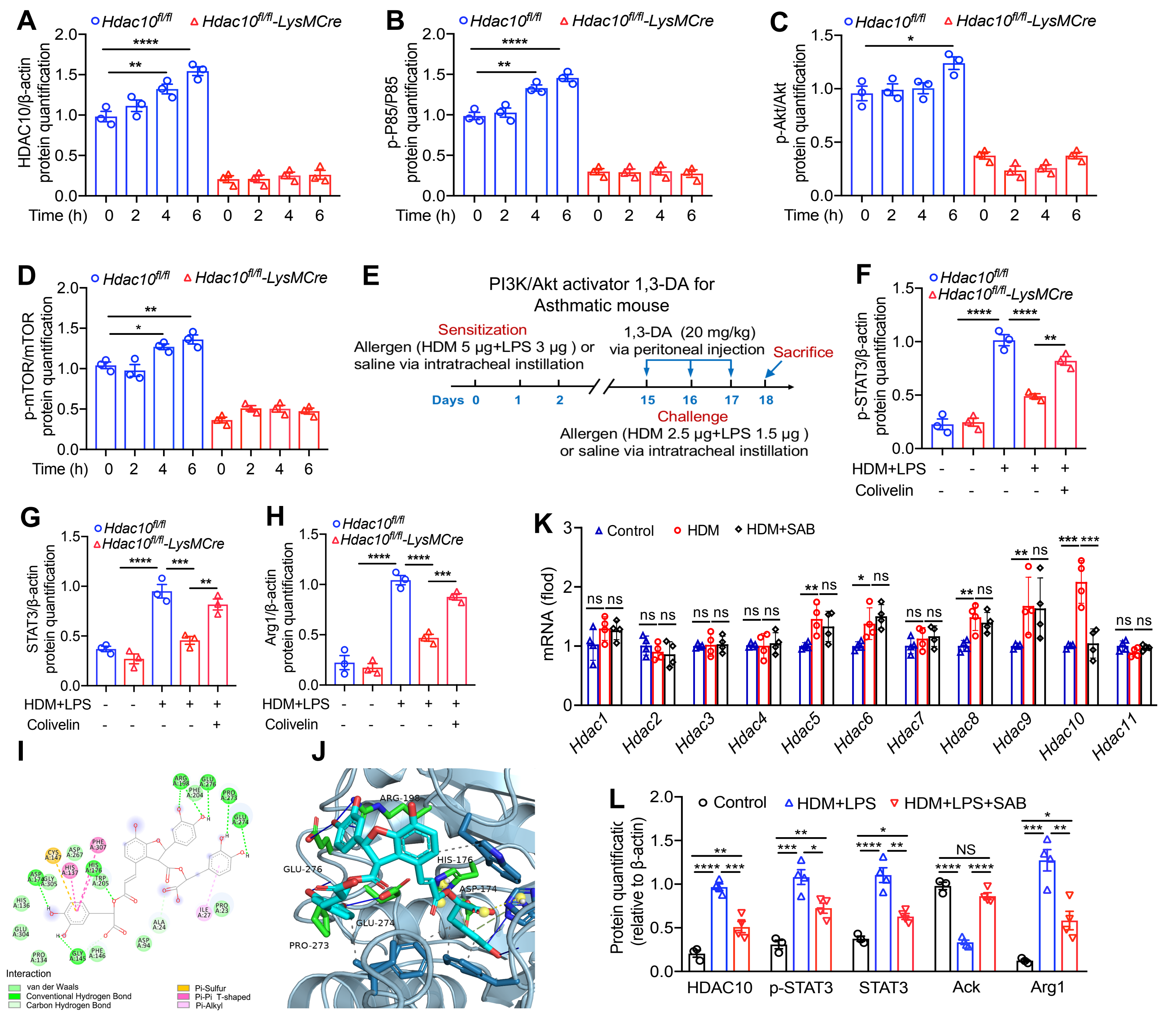
A
Hdac10fl/fl
Hdac10fl/fl-LysMCre
HDAC10/β-actin protein quantification
Time (h) 0 2 4 6 0 2 4 6
B
p-P85/P85 protein quantification
C
p-Akt/Akt protein quantification
D
p-mTOR/mTOR protein quantification
E
PI3K/Akt activator 1,3-DA for Asthmatic mouse
Sensitization
Allergen (HDM 5 μg+LPS 3 μg) or saline via intratracheal instillation
1,3-DA (20 mg/kg) via peritoneal injection
Sacrifice
Days 0 1 2 15 16 17 18
Challenge
Allergen (HDM 2.5 μg+LPS 1.5 μg) or saline via intratracheal instillation
F
p-STAT3/β-actin protein quantification
HDM+LPS - - + + +
Colivelin - - - - +
G
STAT3/β-actin protein quantification
H
Arg1/β-actin protein quantification
K
Control
HDM
HDM+SAB
mRNA (flod)
Hdac1 Hdac2 Hdac3 Hdac4 Hdac5 Hdac6 Hdac7 Hdac8 Hdac9 Hdac10 Hdac11
I
Interaction
van der Waals
Conventional Hydrogen Bond
Carbon Hydrogen Bond
Pi-Sulfur
Pi-Pi T-shaped
Pi-Alkyl
J
ARG-198
HIS-176
GLU-276
ASP-174
GLU-274
PRO-273
L
Control
HDM+LPS
HDM+LPS+SAB
Protein quantification (relative to β-actin)
HDAC10 p-STAT3 STAT3 Ack Arg1
NS

Fig. S5

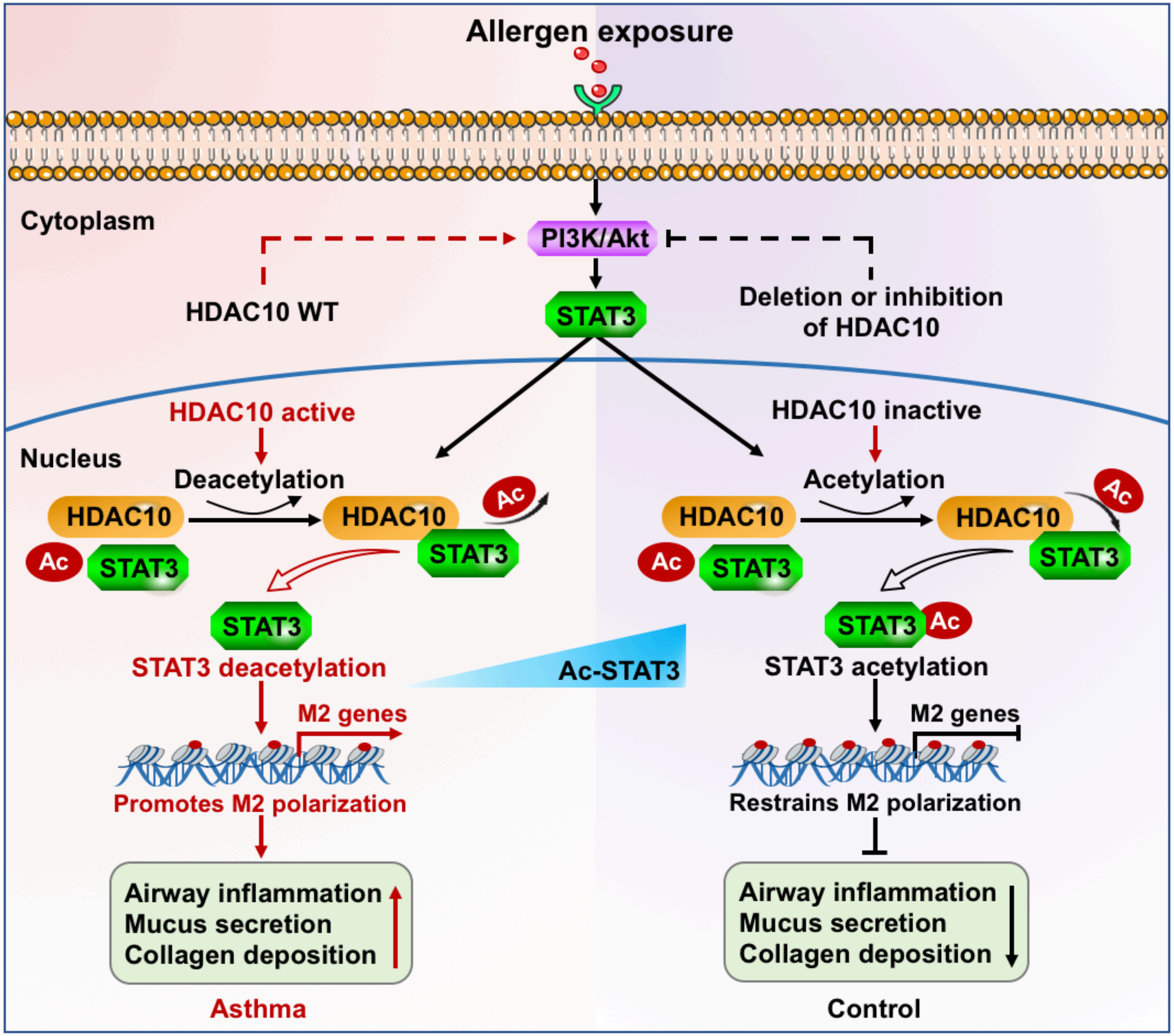

Allergen exposure
Cytoplasm
PI3K/Akt
HDAC10 WT
STAT3
Deletion or inhibition of HDAC10
HDAC10 active
HDAC10 inactive
Nucleus
Deacetylation
Acetylation
HDAC10
Ac
STAT3
STAT3 deacetylation
Ac-STAT3
STAT3 acetylation
M2 genes
Promotes M2 polarization
Restrains M2 polarization
Airway inflammation
Mucus secretion
Collagen deposition
Asthma
Control
